# Perceptions of faculty and medical students regarding an undergraduate research culture activity in Myanmar: a qualitative study

**DOI:** 10.3352/jeehp.2025.22.33

**Published:** 2025-10-27

**Authors:** Htain Lin Aung, Moe Oo Thant, July Maung Maung, Ye Hlaing Oo, Thin Thin Toe, Hla Moe

**Affiliations:** 1 Heidelberg Institute of Global Health, Heidelberg University, Heidelberg, Germany; 2 Department of Medical Education, University of Medicine Mandalay, Mandalay, Myanmar; 3 Department of Preventive and Social Medicine, University of Medicine Mandalay, Mandalay, Myanmar; 4 Department of Mental Health, University of Medicine Mandalay, Mandalay, Myanmar; 5 Department of Pathology, University of Medicine Mandalay, Mandalay, Myanmar; The Catholic University of Korea, Korea

**Keywords:** Undergraduate medical education, Perception, Qualitative research, Curriculum, Myanmar

## Abstract

**Purpose:**

This study explored the perceptions of faculty members and third-year medical students regarding the research culture activity (RCA), a program designed to engage undergraduates in research at the University of Medicine, Mandalay, Myanmar. It aimed to identify the knowledge, attitudes, and skills (KAS) gained, the challenges encountered, and suggestions for improvement.

**Methods:**

This qualitative study employed 4 semi-structured focus group discussions with 17 third-year medical students and 16 faculty members who participated in the 2020 RCA. Student responses related to KAS were analyzed using a deductive framework approach, while challenges and suggestions were examined through inductive thematic analysis. Discussions were audio-recorded, transcribed verbatim in Burmese, translated into English, and collaboratively coded using Atlas.ti version 9.0.5.

**Results:**

Participants reported improved understanding of scientific literature, greater responsibility, strengthened teamwork, and enhanced practical research skills. Reported challenges included limited research preparedness, scheduling conflicts, inconsistent supervision, financial constraints, and weak coordination with inpatient clinicians. Participants also suggested clearer guidelines, pre-research training, protected time, stronger supervision, and institutional budgetary support.

**Conclusion:**

The RCA provides substantial educational value in developing research competencies and remains a promising, potentially adaptable model for resource-limited settings. Its sustainability will depend on institutional commitment, supervisory capacity, and modest financial investment. Future research should prospectively assess KAS outcomes, compare supervision models and group sizes, evaluate digital workflows for efficiency, and conduct long-term follow-up of graduates’ scholarly activities to build evidence for scalable implementation.

## Graphical abstract

[Fig f2-jeehp-22-33]

## Introduction

### Background/rationale

Research training in undergraduate medical education strengthens evidence-based practice, critical thinking, and professional development. Globally, research exposure is increasingly integrated into medical curricula to promote academic literacy and interest in research careers [1-3]. However, in Myanmar, a resource-limited country in Southeast Asia, undergraduate research remains underdeveloped, with limited institutional capacity and insufficient faculty preparedness. The University of Medicine, Mandalay (UMM) launched the research culture activity (RCA) in 2015 for third-year (paraclinical) undergraduate medical students. The RCA is a cohort-wide, compulsory, faculty-supervised, group-based project embedded in the third-year paraclinical phase. It is implemented as a 2-month block (typically November–December) within the academic timetable. Mentored student teams select or are assigned feasible topics aligned with departmental priorities, conduct basic retrospective or cross-sectional data analyses, and prepare a poster or short paper for a program-wide presentation day featuring formative feedback. Operational details (cohort size, grouping, supervisor allocation, and outputs) are summarized in Supplement 1. This initiative is briefly described in the 2019 program guidebook of Myanmar’s outcome-based integrated Bachelor of Medicine and Bachelor of Surgery (MBBS) curriculum, particularly in the vertical module titled “Research culture and skill” [4]. The RCA’s supervised, group-based structure reflects principles of social constructivism, in which students co-construct knowledge through authentic tasks and guided participation [5]. Supervisory roles parallel cognitive apprenticeship models—modeling, coaching, and scaffolding—that make expert reasoning visible [6]. These theoretical perspectives informed our analytic focus on both learning outcomes and implementation challenges. Despite its positive intentions, several challenges were reported anecdotally by faculty and students during the early years of RCA implementation, yet these had not been systematically studied or documented. This study therefore aimed to explore these perceptions in a structured manner. Understanding how such programs are perceived can inform the development of more effective undergraduate research initiatives in low-resource medical education settings. To our knowledge, this is the first systematic evaluation of an undergraduate research initiative in Myanmar.

### Objectives

This study aimed to explore the perceptions of students and faculty regarding the RCA, focusing on: (1) knowledge, attitudes, and skills (KAS) gained; (2) challenges encountered; (3) strategies to address these challenges; and (4) suggestions for improving RCA implementation.

## Methods

### Ethics statement

Ethical approval was obtained from the Ethics Review Committee of the University of Medicine, Mandalay (ID no., 342/ME/UMM/2020). Written informed consent, including permission for audio recording, was obtained from all participants after they were informed of the study’s aims, minimal risks, rights (voluntary participation, option to skip questions, and the ability to withdraw before anonymization), and the intended use of data (scholarly publication only).

### Personal characteristics of the research team

The facilitators, H.L.A. and M.O.T.—senior faculty members with prior qualitative research training—conducted the focus group discussions (FGDs) with faculty and students. They were not involved in RCA supervision or the teaching of third-year students. Transcription in Burmese (by H.L.A. and M.O.T.) and translation into English (by Y.H.O.) were reviewed by bilingual researchers (J.L.M.M., T.T.T., H.M.). All 6 researchers held MBBS degrees and at least 1 master’s degree; H.M. also held a PhD (Doctor of Philosophy). The team comprised 5 males and 1 female. H.L.A., T.T.T., and H.M. had international training in medical education; H.L.A., M.O.T., and J.L.M.M. were experienced qualitative researchers; and Y.H.O., a certified pathologist, was also an accredited English-language expert.

### Relationship with participants

No pre-existing supervisory relationships existed between the facilitators and the participants.

### Theoretical framework and methodology

This qualitative study followed a constructivist approach [5], emphasizing the centrality of participant experiences in generating meaning. In addition to constructivism, the study drew upon cognitive apprenticeship theory [6] to interpret supervisory roles (modeling, coaching, scaffolding) and situated learning/communities of practice theory [7] to situate the RCA within its institutional and clinical environment. These frameworks informed both data collection and interpretation. A deductive framework was used to analyze student responses related to KAS, while inductive thematic analysis was applied to explore broader themes related to challenges and suggestions [8,9].

### Participant selection

Because the RCA was implemented only for third-year students in 2020, purposive sampling was used to include the entire cohort and their supervising faculty as the most relevant participants for the study. A total of 33 individuals participated: 17 third-year medical students (2019–2020 academic year) who took part in the RCA 2020, and 16 faculty members from the Departments of Pathology, Pharmacology, and Microbiology who supervised the same cohort (Table 1). Students were selected across the 3 subject areas and participated in both poster and paper presentations. Faculty participants held various academic ranks, including demonstrator, assistant lecturer, lecturer, and associate professor. There were no refusals or dropouts.

### Setting and context

All 4 FGDs were conducted at the Department of Medical Education’s meeting room at UMM during August and September 2020. Only researchers and participants were present during each session. The setting was private, easily accessible, and conducive to open discussion.

### Data collection

The research team developed a semi-structured interview guide (Supplement 2), which was pilot tested. Each FGD lasted between 60 and 90 minutes. All sessions were audio recorded, and researchers took concurrent field notes. Two researchers (H.L.A. and M.O.T.) transcribed the recordings verbatim in Burmese, which were subsequently translated into English by co-author (Y.H.O.). The transcripts and translations were cross-checked for accuracy by co-authors (J.L.M.M., T.T.T., and H.M.). Data saturation was achieved after the fourth FGD (Dataset 1).

### Data analysis

Three researchers (H.L.A., M.O.T., J.L.M.M.) independently coded the translated transcripts. A dual-analytic approach was employed. For student responses related to KAS, a deductive framework approach was used to organize data into 3 predefined categories: knowledge, attitude, and skills. Within each category, subcodes were developed inductively based on participants’ responses. For the remaining open-ended questions addressing challenges, coping strategies, and suggestions, inductive thematic analysis was conducted following the 6-phase framework by Braun and Clarke [9]. Coding and theme generation were performed manually and supported by Atlas.ti ver. 9.0.5 (MacOS and Windows; https://atlasti.com/). The coding framework was refined collaboratively through consensus meetings, supported by analytic memoing to document coding decisions and track the evolution of codes and themes, thereby creating an audit trail. This process enhanced transparency and reproducibility. Although participants did not review the coded transcripts or findings, credibility was strengthened through triangulation across multiple coders and data sources. Two additional researchers (T.T.T. and H.M.) independently reviewed the coded outputs and finalized the thematic structure through consensus.

## Results

A total of 12 themes were identified from the focus group discussions with faculty and students. These were categorized into 2 overarching domains: (1) KAS gained from student groups (3 themes) (Table 2), and (2) challenges, coping strategies, and suggestions for improvement (9 themes). Themes emerged consistently across student and faculty groups, with several areas of concordance and complementary perspectives. Illustrative quotations for each theme are presented in Table 3 and extended quotations in Supplement 3. The narrative below focuses on thematic synthesis across domains.

### Knowledge, attitudes, and skills gained

#### Theme 1: knowledge acquisition

Students reported a clearer understanding of research structures and terminology, along with improved ability to read journal articles, interpret data, and contextualize evidence within clinical learning.

#### Theme 2: attitudinal shifts

Participants described greater responsibility and motivation, enhanced teamwork, and an emerging recognition that research is an integral component of professional identity.

#### Theme 3: skill development

Participants highlighted a wide range of practical competencies grouped under the category of “skills.” In this context, skills denote observable capacities that students developed through the RCA, beyond knowledge and attitudes. Reported skills included technical abilities (e.g., using Excel for data management), communication skills (e.g., delivering presentations and engaging in professional dialogue with faculty and clinicians), and interpersonal skills (e.g., teamwork, leadership, and precision in collaborative tasks). Students regarded these competencies as foundational to academic and clinical success.

### Challenges and suggestions for improvement

#### Theme 4: student participation

Large groups decreased accountability and added time burdens to private study.

#### Theme 5: research readiness

Gaps in technical skills and unclear guidance impeded progress; both groups favored standardized orientation and training.

#### Theme 6: financial constraints

Student-borne costs were common; faculty advocated for a formal institutional budget line.

#### Theme 7: scheduling conflicts

The RCA clashed with clinical rotation timetables; suggested remedies included protected time and curricular integration.

#### Theme 8: supervision styles

Supervisor engagement varied; students preferred senior mentorship, while faculty proposed more distributed, less hierarchical supervision.

#### Theme 9: coordination with clinicians in hospital settings

Hospital workload and communication barriers complicated data collection; designated clinical liaisons and formal notification systems were suggested.

#### Theme 10: data management

Sample-size feasibility and data quality were concerns; retrospective designs were viewed as more workable.

#### Theme 11: logistics of the RCA presentation event

Limited preparation time, unequal distribution of participation certificates, and unclear marking were noted; proposals included brief pre-event preparation windows, publishing student work, and stronger peer Q&A.

#### Theme 12: digitalization

**Fig. 1.**
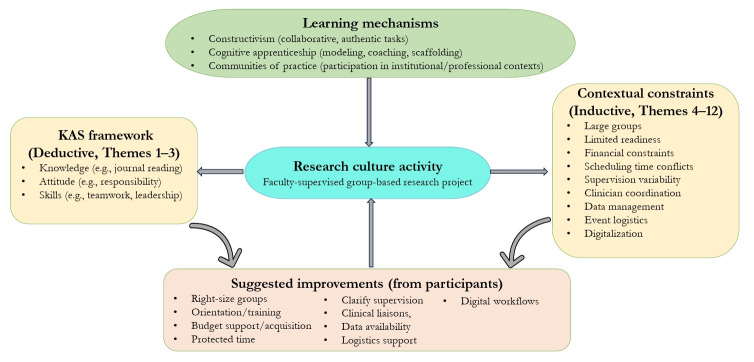
Conceptual/thematic map of the research culture activity: learning mechanisms (constructivism, cognitive apprenticeship, communities of practice), outcomes (knowledge, attitude, and skills, KAS) (Themes 1–3), contextual challenges (Themes 4–12), and participant-suggested improvements. Arrows indicate relationships and feedback loops.

Digital workflows were recommended to reduce printing costs and to build an RCA archive. While many participants emphasized the RCA’s educational benefits, others expressed frustration with large group sizes, repeated revisions, and inconsistent supervision—highlighting the ongoing implementation challenges. Cross-thematic analysis revealed shared priorities and clear opportunities for program enhancement. Despite role-specific perspectives, overlapping concerns and proposed solutions reflected a mutual investment in strengthening the RCA’s structure, relevance, and sustainability. Table 3 summarizes how the 12 themes were constructed (analytic route, primary voice, codes, and representative quotations), while Fig. 1 presents a thematic map linking learning mechanisms, KAS gains (Themes 1–3), contextual challenges (Themes 4–12), and suggested improvements.

## Discussion

### Key results

The RCA supported student development across KAS, while also revealing persistent implementation challenges. Issues previously noted informally in earlier RCA cycles—most notably time conflicts—were systematically confirmed in this study. Understanding was deepened by documenting both student and faculty perspectives. Faculty accounts closely mirrored those of students, and both groups proposed program-level improvements, including structured preparation, clearer supervision, better scheduling integration, and dedicated budget allocation.

### Interpretation

Grounded in constructivism, the observed KAS gains reflect knowledge-building through authentic, collaborative inquiry. Variation across groups corresponded to cognitive-apprenticeship processes (modeling, coaching, and scaffolding) that were applied with uneven consistency among supervisors. These patterns align with the RCA’s intended principles of supervised, problem-oriented learning in medical education (see Results for detailed examples). To sustain impact in low-resource settings, institutional mechanisms are needed to address student preparation, supervision, time allocation, and access to modest resources (including digital support). These insights can inform the design of structured, curriculum-integrated research modules in similar contexts, offering a context-sensitive and adaptable approach to early research engagement in medical education. For detailed skill examples and case illustrations, readers are referred to the Results section.

### Comparison with previous studies

Across diverse contexts, undergraduate research programs consistently promote motivation and skill development but face ongoing challenges related to supervision, time, and systemic constraints. In Oman, both students and supervisors reported benefits alongside heavy supervisory workloads [10]; in a US program, structured mentorship and protected time emerged as key enablers despite logistical barriers [11]. Studies in Saudi Arabia similarly identified structural obstacles—particularly limited time and mentorship shortages—which closely parallel the challenges reported by our participants [12]. In Tanzania, mentorship capacity was found to be a critical determinant of undergraduate research engagement, underscoring the importance of supervisory availability in resource-limited environments [13]. Sudanese medical students likewise expressed strong research interest while encountering practical limitations such as insufficient preparation and inadequate institutional resources [14]. Collectively, these studies affirm that undergraduate research initiatives enhance motivation and skills, yet sustainability is often constrained by supervisory burdens and systemic limitations. Our study extends this literature by providing a combined analysis of faculty and student perspectives within a single institutional context, adding granularity to the understanding of how research culture initiatives can be adapted in low-resource medical education environments.

### Limitations

This single-institution study limits general transferability. The focus group design may have introduced social desirability and hierarchical influences, and participants did not review the transcripts or findings for validation. The KAS component followed a predefined framework, which may have constrained the emergence of novel themes. Data were self-reported and were not triangulated with RCA outputs, grades, or supervisor assessments (these were not collected). Moreover, the qualitative design did not allow quantification of theme prevalence. The feasibility of certain recommendations must be interpreted in light of Myanmar’s political and resource constraints during the study period. Finally, as data collection preceded the post-2022 diffusion of generative artificial intelligence (AI), future research should incorporate outcome-based measures and assess AI-related access and competencies.

### Generalizability

While context-specific, the findings may be adaptable to other resource-constrained medical schools if key conditions are met—such as appropriate group sizing, preparatory training, structured supervision with protected time, modest funding, clinical liaison support, and digital tool integration. Accordingly, broad generalizations should be avoided. Instead, we propose the RCA as a context-sensitive model that warrants local piloting, iterative adaptation, and evaluation.

### Suggestions

Future RCA practice should prioritize the development of clearer operational guidelines, implementation of pre-orientation and data management workshops, and strengthening of faculty supervision through targeted training and cross-departmental collaboration. Institutions can further enhance RCA effectiveness by adopting digital platforms for supervision and coordination and by integrating proposal-writing and research funding literacy modules to prepare students for sustained research engagement. These measures would help address the barriers identified in this study and enhance RCA’s immediate feasibility in resource-limited settings.

### Conclusion

This study confirms the RCA’s value in fostering research capacity and professional attitudes among medical students while highlighting barriers related to supervision, scheduling, and resource availability. The RCA remains a promising and adaptable model for promoting a culture of research in resource-limited medical schools. To strengthen its practical application, future studies should prospectively assess knowledge–attitude–skill outcomes, compare supervision models and group sizes, evaluate protected-time scheduling with modest financing, and explore digital workflows and long-term follow-up to determine whether early exposure translates into sustained research engagement.

## Figures and Tables

**Figure f2-jeehp-22-33:**
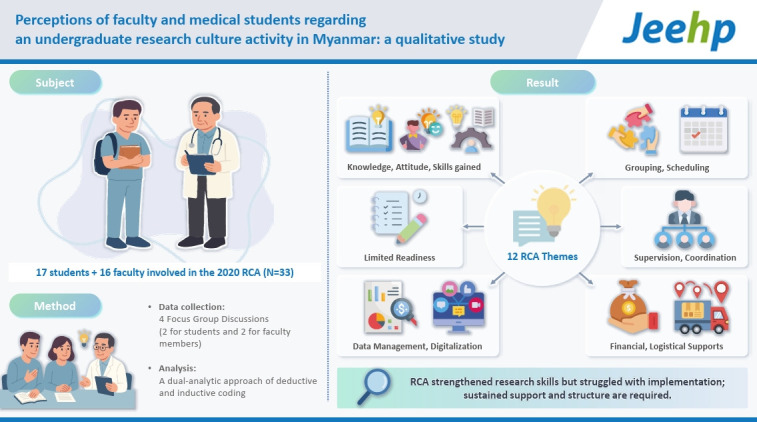


**Table 1.** Characteristics of focus group participants (total=33)

Group	Male participants	Female participants
Faculty FGD group 1 (FFGD1)	M1 (Patho), M2 (Patho)	F1 (Patho), F2 (Micro), F3 (Micro), F4 (Micro), F5 (Phar), F6 (Phar)
Faculty FGD group 2 (FFGD2)	M1 (Patho), M2 (Phar)	F1 (Patho), F2 (Patho), F3 (Phar), F4 (Phar), F5 (Micro), F6 (Micro)
Student FGD group 1 (SFGD1)	M1 (Micro), M2 (Micro), M3 (Phar), M4 (Phar), M5 (Phar), M6 (Phar)	F1 (Phar), F2 (Phar), F3 (Micro)
Student FGD group 2 (SFGD2)	M1 (Micro), M2 (Micro), M3 (Micro), M4 (Patho), M5 (Patho)	F1 (Patho), F2 (Patho), F3 (Patho)

Grouped by faculty and student participants across 4 FGDs. FFGD1 and FFGD2 each included 8 participants; SFGD1 included 9 participants; SFGD2 included 8 participants. FGD, focus group discussion; Patho, pathology; Micro, microbiology; Phar, pharmacology; M, male participant; F, female participant.

**Table 2.** Knowledge, attitudes, and skills gained from the research culture activity: coding from the students’ discussion (SFGDI and SFGDII)

Themes	Codes
Knowledge (K)	New knowledge
	Scientific journal reading
Attitude (A)	Taking responsibility
	Motivation
	Team collaboration
	Importance of research
Skill (S)	Self-learning
	Social skills
	Experience of performing research
	New professional communication experience
	Skill of precision
	Leadership
	Individualization
	Data presentation

SFGD, student focus group discussion.

**Table 3.** Method-integrated synthesis of themes, analytic route, primary voice, codes, and representative quotes

Domain	Analytic route^a)^	Primary voice^b)^	Theme	Illustrative codes	Representative quote
KAS	D	Students	Knowledge acquisition	Journal reading; terminology	“We got to learn new names and Google them…”
			Attitudinal shifts	Responsibility; teamwork; motivation	“We each must take this seriously… It teaches responsibility.”
			Skill development	Excel use; presentations; communication	“I was asked to draw charts and tables… I googled and studied the steps.”
Challenges and suggestions	I	Both	Student participation	Large groups; unclear roles	“A group of 41 students is too large.”
			Research readiness	Lack of training; topic mismatch	“Excel presented great problems… some training needed.”
			Financial constraints	Student costs; no budget	“There should’ve been a budget from the university.”
			Scheduling conflicts	Clashes with rotations; integration	“Include RCA as a vertical module in the curriculum.”
			Supervision styles	Excessive revisions; variable engagement	“Teachers revised a lot—up to 7 times.”
			Coordination with clinicians	Communication barriers; workload	“The hospital should have a person for student affairs.”
			Data management	Sample size; retrospective data	“We ended up case-hunting at outpatient department.”
			Logistics of the RCA presentation event	Preparation time; marking clarity	“Reserve 3–4 days before the event for preparation.”
		Faculty	Digitalization	Reduce printing; archive	“They can share e-books; attendees should receive a copy.”

KAS, knowledge, attitude, and skills; RCA, research culture activity.

^a)^D=deductive (framework based KAS); I=inductive (emergent challenges/suggestions). ^b)^Whose perspective predominantly surfaced for the theme; “both”=students and faculty.

## Footnotes

**Data availability**
Data files are available from https://doi.org/10.7910/DVN/F4DDEQ Dataset 1. (A–D) Raw data of FGD transcripts for faculty and student groups.

## Supplementary materials

Supplement 1. RCA program snapshot (UMM, 2019–2020).

Supplement 2. Interview guide for student and faculty FGDs.

Supplement 3. Illustrative quotations (Theme 1–12).

Supplement 4. Audio recording of the abstract.
