# Supplementary material for: Perceptions of faculty and medical students regarding an undergraduate research culture activity in Myanmar: a qualitative study
Source: J Educ Eval Health Prof. 2025 Oct 27;22:33. doi: 10.3352/jeehp.2025.22.33 (PMC12768548; doi:10.3352/jeehp.2025.22.33)
Supplement: Supplementary file 1 [file jeehp-22-33-dataset1.docx]

**FGD Faculty 1**

Question 1: Challenges related to Supervision, How to overcome, and Suggestions

Answer :

Dr ******

We form groups and supervise them. Not everyone is willing to participate, so we have to play the ‘attendance’ card. And we assign them specific duties, seeing that not all of them are eager to be involved. That means we designate functions by group, like data collection, analysis, presentation and so on. There are some who show up once and never again after; they need constant impetus. Most of the time we have to help them along, since they don’t know how to analyse data properly.

We’re resorting to attendance for the time being so as to get them involved in their RCA (Research Culture Activity). We have to pique their interest, too! Some are curious by nature while others are not, as is expected of students. When the group has activist types, they naturally take the lead. Each group’s responsibilities are distributed among some thirty of them; that means not enough room there for everyone to participate. Also, when the RCA is too close to the exams, some students just choose to focus on the studying. Those looking to get good grades won’t be too keen on the activity. Leaves one wondering if it’s a good idea to revise the timetable so they won’t clash.

Dr ******

We’re supervisors for students put into groups of 35. Not the best of students mostly. We get a mix of achievers, underachievers, eager beavers and otherwise. So we need to be receptive; we can’t just cherry-pick. I try communicating that we are to meet on a specific day when all of them must be present and accounted for. They do show up. That one day and not after. We divide them into subgroups tasked with different duties. From previous years’ experience, we approach the grouping process in such a way that booksmart students belong to the literature review group, the most extroverted become presenters and close friends belong together to literature review, IT and presentation respectively. This is how we allocate tasks.

Such distribution of labour isn’t so bad. Five subgroups work well too. Even then only a member or two from each group appear and the rest after their part won’t show anymore. Coercion using attendance, if I may say so. No amount of it seems to suffice; we won’t actually take decisive action and they aren’t exactly known for regular attendance in the first place. Anyway this leaves us with five or six active participants out of thirty. So how do we overcome that? We can’t realistically expect to interest all thirty. In a way, groups of thirty are too large. Sets them thinking some other guy would do it. There’re roles like that of a factotum: more streetsmart members could help with the typing, delivery of books and stuff. We do our level best to get these students to carry out their research culture activity.

Moreover, we collect attendance on Research Culture Day and keep a record of the data, to be reviewed in case of moderation in the final exams. This has already been announced beforehand. It has also been mentioned by the professor that participation in RCA scores them two points for the final exam. Students are enticed into the culture in this manner. And the merits, if need be, will be considered.

The groups of thirty cannot be made any smaller in the year(s) to come, given the present student-teacher ratio. Presentation is a one-day event, making it even less feasible to feature all thirty groups of ten. So will it go on in the near future, I suppose. The widespread apathy. Even if no efforts go into the research activity, one could certainly do the odd jobs as they arise, refreshments for example. No hands can possibly stay idle, the way I see it.

Dr ******

Regarding the supervision, they come for the attendance, earlier in the process. We take the opportunity to place them into subgroups according to their strengths. They’re enthusiastic enough on that day – data guy, computer guy, analyst, typist, info-collector, etc. Regardless, the last one standing as it turns out mostly is the presentation squad. For shame or for honour, the onus to appear on stage is on them, and so too is the better part of the workload irrespective of the initial understanding. This is one challenge often encountered.

So about what we could plan next. Seeing as how students find the part-1 trip exciting, RCA, a similar event at third-MB level, could be integrated into the syllabus modules introduced at the start of the academic year. That way they won’t be able to come up with excuses citing exams. In addition, groups should be defined well in advance and the curriculum plan laid out early in the academic year. This will presumably solve the current complications and bolster student motivation.

Dr ******

Students can’t make it every time we call a meeting. They can only come on their breaks from the clinical teaching schedule. We only have full attendance on day one. The reasons behind their absence are that the activity is not part of the grading and it is too close to the exam. It was way too close for comfort last year. Actually we have only four or five active participants per group, and it’s all the worse when we have to supervise a group of students who aren’t exactly computer-literate. We even get the impression sometimes that they take it in turns – it’s never the same students. As for the data collection, we provide them with a format pro forma and charge each student with recording twenty sets of data. But we get wind of some students fabricating parts of their reports: they just duplicate some data sets without actually collecting all twenty of them. Another major issue is they don’t know their way around computers that well, which means all related tasks fall to ALs and demonstrators. The tallying is all they do; as a result data processing and other parts are left entirely to us. Even during the printing process they typically take a cavalier attitude to the choice of colours, which would come out too casual, bold or garish were we not there to supervise the whole thing.

So we categorize the oft-encountered difficulties. And the strengths, too. We work like that. Preferably the RCA shouldn’t be anywhere near exams. Even the presenter, the only one that takes an active interest, decides to appear close to the research culture day. This has happened a couple of times. It’s as if to say the others should be doing their parts since his/her role is only in presentation. The rest of the group, on the other hand, seem to hold their own views – that teachers only recommend one of the best for the role of presenter, or something like that. We have to motivate them saying this will come in very useful and all that on their way to MSc. Lastly, even if RCA cannot be integrated into a module, it’ll be more convenient, I think, to make this part of classwork if possible.

Answer : V

How it works is we make it a point to first record their contact info including phone numbers considering those disappearing acts from the students of the previous years. When they do disappear on us, we try to reach them individually. We make sure at least two reliable students are named to each subgroup that is to handle their fair share of the workload. These are the ones that actually do the work when others have simply bailed out. The activity is supposed to be all-inclusive but has yet to include all. So to boost motivation I often treat these diligent students to some refreshments I’ve bought. It was nothing special really, but I reckon they deserve some token of appreciation after shouldering all the hard work their classmates won’t do.

For the years to come, I think it’d be better to form the groups from the outset. Only then will they have a sense of belonging to their groups and the esprit de corps that comes with it. That kind of rapport makes working easier among peers, spicing up the competition.

Dr ******

I’m responsible for assembling the groups according to the clinical timetables in rotation. One issue here is half of the SU1 students are now with the SU2 group, which affects the team spirit to some extent. They have to be brought together by a convincing explanation of how this wouldn’t matter. At the briefing they are notified of the mandatory presence on the data collection day and the first day after grouping of all the members before their supervisor for a subject matter breakdown regardless of prior research exposure. They’re explained what to do today, when to present the paper and how to do it then. If they don’t make the meeting, we call them up and even contact the parents if necessary. So during my five years’ time, I have full attendance on day 1 and presentation day.

On day 1, we focus on motivating them. All of the members must be present on the day of data collection, for which the subgroup for data are primarily involved. They are asked to choose two leaders, male and female, among themselves. Afterwards they are provided with templates and given detailed instructions on how best to collect the data required. Finally the presenter and the co-presenter are chosen. All thirty members involved are motivated in this way.

The subgroup for data is also asked to photostat the format for all three subgroups. This involves all of them in the process and works for the attendance as well. We accompany them when they collect data. The supervisor has also met up with the hospital authorities. This is what our department does. Frankly I don’t want it this way. I want the students themselves to meet whoever’s concerned. On the day of data collection, we supervise them but actually sit and observe what they do. The hospital data are not comprehensive, but the last four years worked as we collected the pre-existing data. It’s not the case the year before that, when we picked up daily data for two weeks. My suggestion here is retrospective studies mostly prove convenient compared to cross-sectional ones that proceed in real time. This is because students are not always reliable, doing things off and on.

Another thing we’ve noted is the 300-bedded Hospital can be ideal for vaild research data. There is no need to fabricate on the part of the students, which is good for our conscience. Things didn’t work so well last year, when the study site was the Respiratory Ward. Patients were few and students have a limited scope of knowledge. Anyway at orientation they have an idea of what to do. With literature review, IT help and presentation, they get around to more studying, about half of the members have a clear overview of what research is like, and the other half have first-hand experience of the data collection process. All in all, they are now able to share information on research procedure, at the same time scoring points for classwork.

It would make things easier if the Dept of PSM could give a lecture at orientation. They did that in previous years. I went to the Dept of PSM with a request, and they explained to the students how to write an international paper. Only then could they understand. What we do to give them insights is we ask them to download papers and explain these and to read up on the study design concerned, hoping this should be enough. The students last year studied all these on their own. I had to help out a lot the year before. Can’t say I’m not annoyed. We had things easy enough, though, four years out of five.

Then the matter of being sharp. It is not just the best students that are sharp; their less-achieving classmates can be, too. What counts here is the quality of being research-minded. The Dept of PSM could make room for a module to this end. We could then set aside a research week, specifically for the data collection. There is no official period for this now; we have to make time from the teaching schedule. Two periods, morning and evening, are cancelled; this can be very draining. We have to be with them all day for the data collection, skipping lunch. For the sake of convenience, our deans could coordinate this with the timetable of the clinical side in order to free up a day or two for this. Two days free of lectures from all three subjects. Otherwise we have to make a formal request for this, only to be turned down later by the clinical side. Frequent requests don’t really make for convenience, that’s all.

Dr ******

There’s no way of telling how many sharp students we get out of the thirty. Sometimes we have only one student doing all the work, which is far from ideal. The key in fact is setting time, a specific time. When it comes to the trip organized by PSM, they have no other academic concerns and are therefore able to commit themselves with a one-track mind. With exams this close as it is, we find ourselves in quite a situation. It’s rather unfair to have only one student slaving away, especially when the exam is at hand.

Regarding the data collection, obtaining outpatient data doesn’t work out well at all for our subject. We have to request permission from the ward concerned, resulting in constant friction when they disapprove. It’s a common problem. Some students actually collect the data with what little time they can spare. Everyone has to do their bit, but it all comes down to the attitude. Some of them only make a perfunctory effort to get this over with. Their data are far from comprehensive, however in detail they have been explained the role of each particular during the pro forma structuring. That’s just how students are. And the data cleaning is not without wrinkles, either. Some patients’ data cannot be counted. Then there’s the matter of cooperation on the part of certain wards. Students are not treated with courtesy. Even harshly spoken to at times. All things considered, the RCA as a must in the curriculum may resolve the issues.

The teacher has a hand in everything, forced by circumstances to ignore the effect on their learning curve. The paper is compulsory. So we push them to their limits, and if nothing comes of it, chances are we teachers end up doing things for them. While there’re some students equal to the task, some simply can’t rise to the occasion. This can be fixed by the Dept of PSM giving a course on research right from the start of the year. Now it seems they’re going in blind and only doing what is asked of them. And it’ll be to their benefit if they’re better-informed to begin with. It’d bring them ease in data collection, too, if topics and supervisors were set earlier. A week or two’s notice that we have is, in truth, rushing things, and it pretty much explains our current predicament.

Dr ******

Speaking from the standpoint of one of the most junior to supervise these third-MB students. The areas of study chosen for them are more often clinical, like cancers or COPD, only taught at Final Part 1. They must quickly catch up to speed: they first try to get their hands on relevant notes, have these xeroxed and swot up on the subject matter. Enough to makes things difficult for them. From past experiences of supervising at study sites like OG (two times) and GI wards, we had to await OPD days that only come twice a week, for vaginal examination and smears in case of cervical cancer. Considering also the turnaround time, it’s no mean feat to pull this off within a fortnight’s time frame. Last year we tried tracking new data as they occurred in the wards but to no avail given the time constraint. This could only be sorted out after the seniors reached an agreement on the use of the hospital register with its retrospective data.

When we followed the students to the wards, the authorities there denied receiving any formal requests even though the university claimed to have issued them. The students as a result got scoldings and talking-tos during the process. I had to step in to mediate, saying we assumed the paperwork preceded us and showing them a copy anyway. These are some of the kinks along the way.

Another thing to note is the students have these pro forma handouts for data collection, which begs the question – is the intention to familiarize third-MB students with the procedure or to apply their results? I see some advanced topics at this level.

Dr ******

What we do is we culture the swabs taken from their phones and gram-stain these at the department. They show an interest and take part, too. But logistics does not permit us to set up the culturing for all thirty. Next year we’re taking the activity to MGH Lab for variety. And to 550-bedded Hospital Lab, that’s to say collaboration is needed. We have to be there for the introduction, specifying the data and the exact period of study for each group. We even have to drive a long way in our cars and theirs if data collection is to be carried out for diversity at more distant destinations like Patheingyi Tuberculosis Hospital.

Dr ******

First we are to see the MS about the permission to conduct research there. And on the day of data collection we have to be there to explain to the students what each abbreviation stands for, e.g. HT for hypertension. Even when they get 150-200 cases, the data of only about 100 could be used. The students analyse their own data when we’re fortunate enough to have a tech guy in the group. Even then it’s not uncommon to deal with their frequent phone calls. But as luck would have it, we’ve only had to give them instructions rather than do the deed. We mostly give them topics fit for Third MB. It can be quite obvious at presentation whether this is the work of a teacher or that of a student: some are so well-written that they’re way above the level of a student apparently. The Dept of PSM won’t take the orientation anymore; so even though we are not experts, we have to explain as best we can the introduction and the references. Of course we edit the drafts time and again. And the one that takes an interest is none other than the presenter themselves fearing the fall from grace. Their work needs to be extensively revised. Especially clever students. We help them work on their presentation skills.

Dr ******

The official paperwork arrives late, necessitating at least one teacher in a group. When the students propose the data collection, they’re met with an outright refusal. The teacher intervenes showing the formal papers. Even the presence of teachers is not enough without the paperwork. Secondly the students cannot use the computer well enough for data processing. A teacher has to help them along as the analysis is something they cannot do. They cannot even write objectives properly. This perhaps calls for some training under the Dept of PSM. They’re hardly willing or able to come when the exam is drawing near, or when they have to be in the wards for clinical teaching. We see they have a tight schedule. We used to give them research ideas more relevant to Third MB like smoking and tobacco. But we need to avoid being repetitive and have other considerations such as resource availability and reliability. That leaves us in a bind. The fact that there are so many groups makes coming up with a novel idea less likely. Overlapping of research areas do happen, and we have to switch and find another focus. We have quite a few difficulties similar to this.

Dr ******

Essentially we only use original papers for Drug Utilization Study (DUS). Three such papers are selected for the three groups, each of which is further divided into subgroups – the study group, literature reviewers and data analysis/presentation squad. When the time comes for data entry, we call up those responsible. Either an assistant teacher or I must be present for the ‘operation’ to fix the trade names that creep in. This is one of the setbacks seen in data entry, which is otherwise uneventful. Since DUS only focuses on percentage, it’s convenient to stick to the original paper and follow its lead. That is, during data entry we only have the problem of brand names. Students have yet to complete all the chapters covering antibiotics and are unaware of the brand names. A teacher has to be there to explain when they are overwhelmed by the widespread brand-name use. Especially considering the purely generic terminology they are accustomed to in the third-MB course, we have some explaining to do.

Students would like to be independent. Some even request the other departments to choose this type of paper, only to be denied. The subjects are different, and some departments base their research on review articles. We’ve heard certain students say they’d prefer DUS done at the Dept of Pharmacology. We have to tell them this happens because of the obligatory grouping and department posting. And the study design makes all the difference. More complicated in subjects like pathology. They need teachers’ aid. In our case students are given a free rein with tables and pie charts. Their work is only modified a bit when reviewed by a professor. Students’ self-reliance means plenty of imperfections, so clearly evident at presentation that one could tell this is the handiwork of students. We don’t even get involved in discussion; they search on their own why metronidazole or ceftriazone is used in a particular case. So they address the questions on their own and respond on the spur of the moment using the knowledge gained from self-study. This is how every subject should be – without planned questions and fixed answers, and starting with the self-reliance in data entry. They can solve things if they’ve done things, and the reverse is also true. With that in mind I make sure the presenter is involved with the others in three aspects: literature review, data analysis and discussion/conclusion. I tell them right from the start to be a presenter only if one can manage all three. It sure takes efforts. But a short burst of energy only on presentation day could potentially cause embarrassment.

Dr ******

We deal with a different mix of students each year. There’s sometimes a breakdown in communications between the data collectors and the presenters, in which case the latter doesn’t have a good enough grasp of the data as the links are missing. Some presenters do not want the role in the first place.

🡪

Dr ******

We cannot plan much for learning, it seems. Proximity on the roll decides the grouping. Any person to equitably divide the groups has a lot of work on their hands. So we’ve got to take whichever group we are given. It’s just luck whether or not any group enjoys brilliant students. When we have these gems, the workload is eased and the presentation is decent. But a teacher is honour-bound to mentor students of every ilk, not just clever ones. We must get results from any team of students we work with. There’s nothing much to say about this grouping by name or roll number. The real challenge is in the subgrouping, i.e. choosing the roles like that of a presenter or the group rep. The matter is put to the vote amongst themselves. We don’t stipulate the roles; we just let them distribute their own labour. The subgroups are thus formed, through their suggestions and of their own choosing.

Dr ******

When we group them, we first find out which wards they are in at the time. Then what areas are being considered for the research by Pathology, Microbiology and Pharmacology. We should have a proper meeting here, in my opinion. Attended by all three heads of department and some teachers including the group-maker myself. In reality, it’s only my dean and I working, and for that my conscience bothers me. I’d hate for it to look like we’re handpicking the cream of the crop here. Students should have been to both Medicine and Surgery for our subject. This leaves students of the other subjects in sort of a transition between wards. I’m not happy about that. The issue should be addressed by a multidisciplinary negotiation between the three deans and the clinical side. This is putting the solution plainly. Whoever’s in charge at the departments will have to come together and settle this, should the deans be otherwise engaged.

And there should be one clinical representative present for better communication. Those at the 300-bedded Hospital are really open to negotiation. There should be clinical involvement, essentially. Our presence there may be thought to complicate things, and though I haven’t been through this myself, it is experienced commonly enough by some of my colleagues. Take for example the anaesthetists who wouldn’t allow us to refer to their charts. This kind of prohibition has a psychological effect on the students. So all of us, both teaching and clinical, need to embrace this research and be completely receptive. This is where we start. We accept the students. We don’t respond temperamentally at the wards. Then and only then can they grow the faith and drive necessary to accomplish the research.

I pay attention to feedback. It’s often questioned if students cannot conduct research related to the subject of their choice – pharmacology enthusiasts to undertake drug utilization studies, and aspirants for diagnostics to research their field of interest, pathology or microbiology. Now it’s as if they’re forced to do whatever falls to their lot rather than pursue their interest. Ideally each should be asked which of the subjects they are bent on. And also informed of what research on each subject involves doing – the study sites, the topics and so on. They should then get back to us with a decision, after being given some time to think. This is the kind of planning that should go into the research activity. But it’s quite the contrary now. I divide the students into groups, which the other departments take. I don’t know much else. It’s simply that upon meeting with seniors I’m instructed to form groups of students. Convention dictates that we give them topics, and I don’t really feel as though we’re disregarding the students’ wishes.

Dr ******

It matters how much time we have. We’re now planning the event at short notice and when the students can barely make it. The situation calls for the teachers’ intervention, it seems, if we’re to get things done. Here planning ahead would provide them with plenty of much-needed time to think. We cannot however individualize it to such an extent as to allow each student the subject of their choice. So we just have to make the best of the subject we get.

Dr ******

We get all the data collected through the group leader and cannot give feedback on the shortcomings of each member. Time-permitting we should be able to iron out all the rough edges encountered at data collection. We can thus learn the way they deal with one another. It’ll be for the better if we can find more time to spare. Then we could ask them to hand in the data personally. We could also gather students’ feedback each year. That way we’ll be better-prepared in exploring ideas for the next year. From past challenges we can get lots of novel ideas for the years to come.

Dr ******

The students have their heart set on the prize, as they should. The presentation would ‘reinforce’ them. Now what follows naturally is the teachers get involved, aha, vying for the glory, it appears. Indeed some remark in jest that this is way more than a contest between students.

Given the way things are, the time factor allows us little room to demand from students the execution according to plan. They seem awfully relaxed. This makes it hard to stick to the schedule, and we fail to tick things off the to-do list at certain checkpoints of time.

Dr ******

I am something of an interdepartmental liaison. I make sure all the final drafts are ready three days ahead of the D-Day. Each group, poster or paper, is to finish the draft on a specified day, edit and proofread the day after and submit it to the ME Dept one day before the presentation. After an in-department trial run, the students know what parts of the draft need sorting out and become more aware, for example, of how fast they are speaking. For presentation there has to be a draft, and they need a minimum of two practice sessions with me, the supervisor – at least once at the department and more, if necessary, at home.

Dr ******

Time counts. They could barely get the final draft out in time, let alone practise the presentation. The Powerpoint slides also have to be prepared from the completed draft. Only after this can the presentation rehearsal come. The problem we had last year was I only got to listen to my group’s presentation when the others are out on stage. We were in such a rush. Everything, including students coming to meet their supervisors, comes down to the time. We don’t want to take the work home, and students won’t necessarily join us even if we’re willing to.

Dr ******

My house is not within a convenient distance, so on days off I have to train them for presentation at the university.

Dr ******

It’s exhausting for all of us to spare this much extra time, when this is easily solvable by a fixed period of time for RCA. Now they have to devote to it large chunks of their private studying time.

Dr ******

Since the groups are formed, I’ve sent to all the departments the format required, including the referencing style, the font and how to prepare the papers and posters. Whether or not the students are aware of this depends on each department passing on the information.

Dr ******

Yes, there are formats. The fact is one student receives the format, another prepares the paper or poster and yet another one brings this to the supervisor. The format as a result is off in many respects and needs considerable amending. To sum up, it is important that the students are research-minded; the amount of time or supervision is less relevant for that matter. They could do with an orientation workshop, organized by PSM perhaps, which aims to develop research-mindedness in them.

Dr ******

In my opinion the students should be provided with a handout for RCA. For all of them. There’re many portions to research work in practice. Some students have limited knowledge of computers; others, of the font and format. And we have so little time to cover all these. They’re all of a sudden faced with this daunting task without prior capacity-building. It may be a good idea to include this as a vertical module in the integrated curriculum.

Dr ******

Again with regard to the time factor, the RCA does not have a time period set aside, say a week without lectures. Even if they do skip their teaching periods, they can only free up half a day or so. The time is a real constraint. What we also experience is the postponement of the event when they’re all eager and ready, due to a clash in the rector’s or a professor’s schedule. This kind of dampens their enthusiasm, and we have trouble recalling them. Frequent changes to the time schedule inconvenience us. We should have collaborated with the clinical teachers in drawing up the curriculum, if I may suggest. Now we with our ranks cannot just go negotiating on equal terms with the clinical teachers. Just a week free is enough, I suppose.

Also the RCA topics should be commensurate with the students’ capabilities. How far can we go in terms of the scope of research, around which time will the actual data collection take place, and how much of each subject’s course will have been completed by that time? We ought to include in the research only what has already been taught. This is not the case now. Some areas are compulsory while the teaching is only halfway there. It takes a lot of efforts on the part of the supervisor to rush through the topics yet to be taught in class. The students also have the problem of presenting a research topic without quite comprehending it first.

Dr ******

Our department is responsible for the budgeting, among many other things to do. First we have to collect money from all the departments, since we are to stand all the expenses. Ours is not a department with deep pockets. But we would like to award the outstanding groups, and for all those who have worked hard we have consolation prizes to confer. They feel rewarded when they can treat themselves to something with the prize money. The rector contributes what he can, sometimes half and sometimes a third. We cannot however take this for granted, and so we have each department chipping in. it’s our department that estimates the projected amount to collect. We must decide on a ballpark figure that is neither too little nor too much. Since we’re responsible for the financial side of the competition, we have to manage and set the cash prizes referring to the previous year. We consider the gifts for the judges as well. That is a long list of expenditure, not counting the refreshments which the students arrange. It is more or less a burden on us as a department, a financially challenged one at that.

My suggestion here is, it’d be better if the university provided support. The UMM Alumni does lend some financial support, but we cannot count on it. If possible, a specific budget should be included under Third-MB Research Projects, whatever the grant is or from whichever level it may come, MOHS or the rector himself.

Dr ******

As far as I know, we proposed a budget for RCA the other year. Some teachers drew up a plan, but it was never responded in kind. That should have come to something, considering the huge budget surplus that goes back into the government’s coffers. There should be a budget allocated towards the university as a whole, if not separate departments. The undergrads deserve their due when research funds are readily approved at postgrad level.

Dr ******

The expenses, excluding the refreshment costs met by students, added up to K 1,400,000. Upon calculating the amount to be afforded, we were instructed by Rector to consider four parties, his own office included. Sometimes only two parties, with the rector’s office bearing half the expenses and the three departments sharing the other half. It went that way for three years, and the four-portion system has only been used for the last two years. The 1.4 million covered all the costs, from the prizes to the gifts.

When students meet up with us, we ask about the costs incurred during the data collection. As a show of courtesy we have to tip the orderlies and gift the authorities at the ward. The students also do this, but it’s on them. They raise a fund of their own to get necessary documents copied, to have the books printed and bound, and so forth. And for the refreshments, they have to come up with enough cash. We could find them sponsorship one year, from a jeweller’s. And from Soe Soe Myanmar Noodles the following year. This and last year they had to spend, K 5,000 each. The money was used to serve lunch to the attending teachers and students themselves.

It usually costs K 3,000-5,000 per student, coming out somewhere in the region of 1.5 million kyats for a class of 400-500 students. This and the funds for prizes make a total expenditure of roughly 3 to 3.5 million kyats.

And complaints from students always come. There was one student who even said “we have to pay for the food you eat”. That’s enough to make a teacher shy away from telling these students to raise the money. We can take care of all things research-related. But not stuff like “we give prizes and you take the catering” anymore.

Others Issue

Dr ******

The RCA involves third-MB students, so needless to say the judges on the panel are pretty forgiving and appreciative. The same cannot be said of the senior teaching faculty. Appreciation is at the centre of medical education, not the blaming. So it can be upsetting. But who’s going to raise the issue? Now the research-naïve student onstage is greeted with admonishments and rebukes. If they do decide to share that kind of experience, willing presenters will be few and far between in the future.

Appreciation rather than apportioning blame is the way to go. As is courteous treatment. Leave them to do what they can. That presenter girl last year needed plenty of soothing and pacifying.

It’s good practice for students to question and answer one another. This has little place for the voice of the teacher. Shooting questions is one thing; blaming is another.

Dr ******

I once supervised a supplementary group and waited out the whole thing. What I mean is it causes the students stress if the teachers leave prematurely and won’t keep a hopeless bunch company. So I wait, even if it’s only for motivation. Any group of students will have their eyes on the prize, and it’s probably wiser to not disclose which team stands last.

Now instead of first, second, third and consolation, it’s fourth, fifth, sixth and consolation.

Dr ******

Only the winning group, the runner-up and the second runner-up ought to be disclosed; then the last three in the standings could remain under wraps. Simply consolation should be mentioned as there’s no first, second or third in consolation prizes. This should be random or in order of the lots they drew for presentation.

Dr ******

The negotiation between the rector and his deans is one thing. Now the Dept of Pharmacology has apparently become the starting point and pivot of RCA. There should be subteams on the committee to initiate, to take meeting minutes and to report. We’ve been, as it were, issuing all the directives. The department heads should be delegating the duties in a meeting with the rector. It shouldn’t be that I’m now the only one who knows and instructs what to do. The teacher being told to carry out a task may have reason to think I am the one calling all the shots.

Done

**Faculty FGD 2**

Q1: Supervision/Challenges/How to Overcome

Dr ******

I’m part of RCA almost every year. From my experience not all the students can be involved. In order to get them all involved, RCA should be considered as a portion of the criteria for passing the students. The attendance right now is our only means of persuasion.

Dr ******

From three years of experience, each of the three departments handles 150 of the usual total of 450 students. Our department assigns more students to the two papers than to the two posters. The poster was prepared by only 20 students the first time around, an active 20 thankfully with the exception of the three repeaters. Those three had to be notified with a phone call to appear for attendance. We rounded up all the students for contact information as their presence is mandatory for the day of data collection. The next years we have 40 students, of which only 30 showed. The usual phone calls for the rest. Being repeaters they have poor contact between themselves, requiring us to ask around. We did try to reach them using the contact number in their student cards, only in vain or sometimes reaching the parents. Students not providing true info gives us a hard time. Then they argue they haven’t been properly informed.

To become all-inclusive, we need enthusiasm on the part of these students. They’ll only do what they think is important. As an extra-curricular activity of sorts aiming for knowledge or experience, not even a third of the students may be willing to participate. We may or may not successfully entice the better students, but one thing for sure is the last-chance students are uninterested. They could be thinking their work is done after the data collection.

Dr ******

For three consecutive years I was responsible for posters by lot. Posters are seen as less of a burden by students. All 30 of them appeared for the first day, and I made groups involving all 30 in data collection and also the subsequent data analysis, poster preparation, literature review and presentation. In reality only five students or so carried out all these tasks and the rest were nowhere to be found. They weren’t really tight-knit either. The team spirit’s missing. As far as I could see they believe someone else will do the job even if they don’t. Consequently all the work from start to finish was done by a conscientious few. Still they cannot be left unattended, resulting in teachers getting actively involved, constantly pushing them and revising their work should they make a hash of the data. As a supervisor in the literal sense of the word, I cannot just leave these students to their devices, and this leaves me with plenty to do.

If we are to improve on the previous years, it is question of just how much of this research work is applicable to the exam grades. An incentive so to speak. Integrated as part of the curriculum, led by ME. This could be enough to persuade them and possibly draw two thirds of the students to RCA, if not all.

Dr ******

First we meet with all 35 members in the group. Actually only ten or so of them are also chosen to do the research work. Some of the rest won’t even show. The members don’t all know one another well and are not aware when one of them goes absent. The group may have hard workers. And also students between the two extremes.

What we do next is we allocate tasks deciding who is to do what. But when the individual tasks come together as a whole, it is not quite of an expected standard. We make frequent phone calls early in the process, but later we form a messenger group to check up on their work and so they can find out who’s in the group and communicate as necessary.

Dr ******

As for the matter of the group size, 40 out of the 45 students attended and five were missing. In that case, we form subgroups of six. Another issue is they are posted to different wards at different hospitals, like MU 1, 2 and 3 of medical wards. Different wards. So for convenience of communication we appoint a leader to each subgroup, whose task is to assure the proper data collection of the seven members and after the process to calculate the data if his group is responsible for the calculation and analysis. The supervisors may then interact with the group through the leader, who relays the message to the members. The system works well enough.

Also we elect two general leaders to the whole group of 45. For our research if not for the event-planning. The photostats, budget management and the trivial matters. We designate co-leaders as well. Students of some batches volunteer to be presenters whereas those of other batches do not. The role is filled if someone in the group steps forward to volunteer. We draw lots to decide on the role when no one does so.

Question About Data

Dr ******

Students do not know much about the important aspects of data collection. A format checklist needs to be set for the process. They must be told what types of data are required of a research on a particular disease. Some students adhere to the format and do a complete job while others enquire no more when certain data are not found on the register. Not all of them are present when we convene a meeting before data collection. Even though on day one we brief them on the procedure, the same students won’t show up the next time. Nor do they properly hand over the duty to their classmates, who go about the data-collecting process in their own way. The orientation should only take place when all the students are present. We compel them to come, but there’re some missing anyway. The roll-call system should probably be used to define the tasks like data collection. Any setback in data collection potentially leads to conflicting interpretations.

Last year we studied the patients at STD Clinic. We came to a dead end in follow-ups since some cases attended only once. Or we were not able to track the cases as the study was over. Many cases were excluded as a result.

I suggest more time is devoted to data collection, more than the two weeks we are given. But it can’t be said to be too short either, given that we have one month allowed for RCA already. All we can do now is to make sure they collect data properly. The problem with this is they are not there to listen. On top of that, the teachers have also to break down the data and ghostwrite the analysis. Sometimes when we ask them to search for relevant journals, some obligingly come up with these and others just make excuses saying the teachers did that for the seniors the year before. They would complain about having to find so many scientific articles. The thing is maybe this comes too early for the students’ capacity to cope. And the data analysis may be too complicated and confusing for them.

Dr ******

Time I think is the root of all problems. Sure there is one month set aside, but not exclusively for this. There are routines like lectures complicating plans. Research is rather difficult by nature, and they can’t seem to wrap their heads around it. This has an impact on everything that follows. We have concerns constantly on our minds. When each student administers ten questionnaires, we feel somewhat unsettled wondering if they actually collect all ten as per protocols. Some of them may be sticklers for standard procedure, but I’m worried there might be others so apathetic as to make up the data. We have serious concerns at this stage over compromised data integrity.

Foremost in the requisites is their interest, followed closely by more time allowance. A common problem is the clinical teachers refusing to cancel their periods, and it comes at the expense of our lectures. Even though we could let go, problems arise when they cannot sacrifice their teaching time on many occasions.

From my experience overseas, students have three months free of everything but the paper in hand, but each must do one and make the most of the three months. They learn a lot as a result. Although we cannot expect the same end goal, we proceed with what we have under the circumstances, which leads us to the present state of affairs. Also, each project involves a minimum of 30, not all of whom are outstanding students. But it must encompass everyone, especially in data collection. The value of certain data has diminished consequently.

And it’s the teachers that come up with research topics based on master thesis titles, which is too high for these undergrads. If ten students take on one topic, then there’ll be many papers and posters. Spread thin enough, we can get more of them involved. Then the topics must not be too difficult. Light and feasible, allowing them more freedom to operate. It’s what I think.

The natural consequence of more teams is more workload. Each team is now led by about six teachers in hierarchical order each supervising one rank below. From my point of view every teacher is perfectly capable without a hierarchical structure of overseeing small projects that only aim to instil a research interest into students. I see no reason why it shouldn’t work to give the teachers more latitude.

Dr ******

I agree with you. We have a sizeable population of teachers here, each of whom can potentially lead a subgroup comprised of the number of students dictated by the student-teacher ratio. In addition we have the clinical teachers proclaiming how their workload precludes them from participating. It happens. These teachers are in fact better able to track the data and would make more efficient supervisors. Accordingly after division of labour in terms of relevance they should take on a portion pertaining to respective areas of clinical research and handle a small number of students, which is not in the least unfeasible.

Next, the topics are only fit for grandstanding. We have a highly competitive culture to be frank, and it’s counterproductive in my opinion. I think it is meant for the students to gain insights into research procedures. Basically to give them a taste of research, the way I see it. This shouldn’t go so far as the them-and-us attitudes between the departments. Individual teachers assuming separate supervisory roles should end this mentality, I suppose.

Lastly I don’t quite like the custom of post-RCA obeisance-paying to supervisors. Not to go against the deep-rooted convention, but the concept of quid pro quo should not exist here. This too, I presume, would be eliminated by the single-supervisor system mentioned above.

Dr ******

There is a slight disadvantage to data collection in that it is extracurricular. The timing is really unfortunate considering the morning lectures are over at 12:30 and clinical training starts at 1pm. When we plan to accompany them on data collection, they claim they have to be at the hospital. From my three years’ experience, the most distant was the TB zone in the industrial township last year, taking over two hours there and back. When they get back in their wards later than 2 pm, they miss the roll call; so not all of them join us, only ten or so as it turns out. They take turns going on data collection, and it is not the same group the next time.

The next issue is the leader having no real influence over the rest of the students. They won’t follow however much the leader may push them. The leader whom they elect themselves, mostly someone with presentation experience during 2nd MB. Not all 30 members have a say, though. Only those ten or so present on the day of election.

For convenience’s sake, the activity ought to be curricular rather than extracurricular. The process need not take place within a month or two in succession; one day weekly set as research day would be fine.

Concerning the choice of research topics we tend to set the data collection sites farther away in our attempt to avoid topic overlapping. This is how we came to choose the TB zone.

Dr ******

We at the Pharmacology Dept study drug utilization mostly. It suits the students’ level. The first of my three years of supervision focused on antibiotics. The data of the surgical wards were collected within two weeks by respective subgroups. What we experienced was the students were willing to procure the data only from the wards they were in. So we divided the subgroups accordingly, provided each with a pro forma and told them the possible brand names of the antibiotics they’re studying. There’re not many antibiotics in use, and SU 1, 2 and 3 were sufficient. The trouble was in the arrangement of the beds: a particular section of the ward had already been allotted, but the recovering patients had to move away from easy-to-reach sections. Moreover there is often a heavy caseload on admission days, and antibiotics likely haven’t been administered to many of these new cases. Thus our instructions must be complete so that students also make a point of checking the postop patients for antibiotic use.

Once, upon a meeting with Head of Surgery (Prof. XXXXX), we after the official notification had to explain all over again before enquiring about the operation days and postop antibiotics data. We had provided the students with comprehensive sets of formats to avoid case overlapping, but that increased the workload for the students. Expecting our exclusion criteria to invalidate a substantial portion of the case data, we had each student report ten cases, and out of the couple hundred samples we eliminate duplicates and discard unreliable data.

Another source we draw on is the retrospective data. Requesting patient charts from the 300-bedded Hospital, we task students with checking for the drug in question. The handwriting is often barely legible, and they’d ask us when they can’t make sense of it. Between the scrawl and the trade name use, even we teachers find this near indecipherable. We show them the general approach – how they are supposed to write the generic and trade names as well as include the dosage, where from the order form they may glean relevant pieces of info and that they should only note down the discharge diagnoses. They’re not very familiar with this.

Dr ******

We also encounter a similar issue regarding data. Even the teachers have a hard time navigating these charts. And the follow-up issue as well. We had previously relied on the hospital registry for our data collection assuming it would be a simple data pickup. But the plan’s upset by a lack of record-keeping. Deeply inconveniencing when students have to get down to it themselves. I say this is on the hospital, which could make the process easier by proper infrastructural support and data archiving system. Additionally the authenticity of the data may be ensured by the inclusion of the clinical teachers.

Then there’s poor communication between students and teachers. We provide them with our contact numbers, those of junior teachers in particular. But they contact us off and on. Teachers differ just as students differ, and there are some teachers that are not actively involved. So the aforementioned idea of duty delegation may yield more. And phone contact is more effective by far. So is utilizing social media as a tool of communication and progress checking. We work at weekends as well, though it poses communication problems to teachers and is less convenient for students. We somehow overcome this urging students on, even at times we are feeling unwell.

In short, the topics made simple plus the teachers spread out would hopefully remedy the situation.

Dr ******

The hospital authorities need to be primed with proper introductions by three or so of the teachers before our students go for the data. On one occasion when they thought this was for a master thesis, we had to thoroughly explain how our university organizes small research projects for undergrads. But it was rather unproductive when the students actually went collecting the data: the data were incomprehensive with only name, age and chief complaints stated, sometimes without the diagnosis or treatments given. Complete information could not be found even in charts, according to the students. This required them to take history straight from the patients themselves, which did not go down well with the medical officer in charge; they ended up resorting to the customary palm-greasing with some gifts they had bought. And there was much, they found, to which those teachers were inattentive.

All

The official notifications for RCA reach only the MS and the high-ranking. They’re the only ones who know. Those personnel of lower rank that students will have to deal with are unaware.

Dr ******

If we’re to study cases at 300-bedded Hospital, the MS and deans need be properly informed by senior teachers rather than junior.

Dr ******

Regarding communication there’s also teachers’ communication to consider in this hierarchical system. Frankly there’s some concern about speaking up for fear of being held accountable should something happen to the group. In the two years I’ve supervised this, I’ve conducted myself with propriety and discipline. I cannot be way ahead of the rest. Not too much self-control either, dreading I might be snowed under. Then the expectations can be lofty as apparently all the departments will be contending for supremacy on research culture day. Pressure intensifying. And when we work small stumbling blocks get in the way. I’m a mid-ranking lecturer. Those junior to me may also have a similar experience. We do not end up supervising as intensively as we would initially like, perhaps fearing some inadequacy would be attributable to them. All eyes will be upon that particular teacher in case of a mishap, responsibility naturally lying with the overseer as seen in precedents. Which brings me back to my point, the postulate that one supervisor per unit works best. The teacher would give his/her best, and the students should learn optimally in this setting. A counterpoint however is we would be spreading ourselves too thin. This will be no issue once RCA is incorporated into the curriculum, no teaching periods meaning immersive experience by teachers and students alike.

Suppose we could reconsider the timing. The study types we’re using have data at their core, hence the importance of structuring and knowing the questionnaires. We therefore set aside one week for questionnaire development and orientation, followed by the resumption of the routine curriculum. We may then specify another week of actual data processing when the RCA is upon us. The approach may have more merits than a single long stretch of time. We might want to work on diferent occasions in terms of pre-data briefing, data collection and post-collection phase. Students are likely to be reenergized when reconvening post-data.

So two occasions, the first aiming to prime the students for the upcoming research directions. Then the actual data-collection process. Another preparatory session right before RCA should be enough.

Self-Learning and Group Learning

This is done with the aim of facilitating students’ critical thinking. So a topic of their own choosing would do well to spur them on. Now with the time limitation we cannot afford to give them much time to ponder over the choice. And when they do pitch in with a topic or two not considered potentially feasible, the teachers must set the topic which may come across as being forced upon them, and the apathy ensues, I guess. Thus the right to pick the research topic may open up more learning opportunities for them.

Dr ******

The fact is it can make things easier if research methodology is introduced in a lecture at the start of the third year maybe. It happens now that they are fed a whole new load of information in a mere month and are at a loss as to what to emphasize or study. When it comes to research and how to write a paper or a poster, they have not the slightest clue about the basic components of a research paper, nor the steps involved.

Dr ******

It is advisable that RCA, when in the curriculum, be reinforced by a series of small assignments in addition to lectures, preceding the major project. Theory alone scarcely imparts research techniques even to postgrad level. Aside from lectures, we can use small-group teaching to assign individual tasks, each focusing on a certain point they’ve learnt in theory, designed in such a manner that presents them with an outlet to practise particular aspects of the methodology explained in the lectures. Nothing too heavy. Just an aid to comprehension. We let them try after taking some time to demonstrate. Maybe this is how we capture their attention in this climate of apathy. Apathy so widespread even postgrads are afflicted, it seems.

Dr ******

We now have to describe what research is like when RCA is about to begin, and elaboration on theory at the time is most often responded by students going ‘how much longer’ or ‘how boring’. We find it really discouraging. Such a response is likely brought on by the intrusion on their spare time. Demonstators like me, just above the students themselves, are not at the best level to explain. But we’re constantly exposed to them. We have good communication. Using this we show them the scientific journals we have printed out and broaden their horizon internationally.

Dr ******

Although we do research relating to third-year subjects, junior teachers of PSM as an introduction could explain the stepwise approach to research, demonstrate how to highlight the key points in a journal and maybe lecture them on literature search at the library. As for the topics, we can get six subgroups out of thirty students and charge them with proposal of research interests. We may then look for a viable option fitting the clinical setting and make suitable modifications. They’ll probably be keen on this type of group learning. And they’ll take the self-learning as far as it can go.

Dr ******

In my opinion the university would do well to adopt a guideline for these presentations, as with the PG. A university should always have a fixed guideline. Then we can bring it to students’ attention. Even if they need not do anything too heavy, there should still be a guideline for smaller projects. We now have a protocol only for thesis. Nothing for posters, the preparing of which more often than not is an unnerving experience. We have to make enquiries at the leading department. A lack of consistency is inappropriate. A format or a template needs to be set. Should save students some time.

Dr ******

They can’t really be self-reliant when preparing for RCA. That weight is mainly carried by the teachers. They can do only so much as data entry. As the preparation process must ideally include the presenter, we choose the team with him or her in it. We want the presenter playing an active role in the steps leading up to the RCA, but the ones who did not get the role by volunteering won’t show interest. Oftentimes, of the two students elected to the stage, the co-presenter is a little less enthused. We can have organizational motivation issues that way.

Dr ******

A format would help. Now we have to refer to previous years’ works for margins and layouts just before poster printout. And redo it if that doesn’t look the same. It costs the students each time. Even if they accept with good grace the drudgery of paper-length posters, the frequency of all the editing and revising can be off-putting.

Dr ******

In paper or poster preparations, it’s always down to the budget. They’re students with no fixed income, but each of the twenty or so students has to pay at least five grand (kyats) for the vinyl posters, book copies and refreshments. Budget redistributed where necessary. An official budget grant would make life easier, seeing as how it’s costing them time, money and energy. The university or the government should develop a budget plan for all related expenses, if I may suggest. This is taking a lot out of the students, and their pockets as well. The total amount, counting the prize-giving, comes to a minimum of 30 lakh every year. The financial burden is shared between the students and the departments. And to a lesser extent I think, the university.

Then the matter of obeisance-paying. It is not something that can be forced. Suppose our group is lucky enough to stand first in poster presentations and is awarded a decent sum. They would use a portion of the cash prize and buy a sarong as a homage-paying gift, that’s all. This would happen.

Dr ******

It’s a common source of contention faced by the group leader that not every one of the forty members is there to contribute to the funds but none would waive the right to their share when the group wins.

Dr ******

A thought struck me the other year. Why does it not occur to them that in this age of digitization printing enough hard copies to go around isn’t really necessary? They may simply share the e-books reserving the handouts only for the senior teachers. Easy and cost-effective. All attendees will be receiving a copy they want, so the system should be seriously considered.

Dr ******

During the RCA season when students enquired, we search the books stored at the department for relevant materials, which isn’t many. We capture these photographically and pass ‘em around. Things would be far more convenient with a research corner. Or a website. Even video documentation of yesteryears’ endeavours. Now students are asking how it was done last year so close upon the rehearsal. Word has it that the rector is planning a research corner. Can’t be sure if that’ll actually happen. I do know we have sent the digital files to the office. No idea though where or how they are stored.

Other

Dr ******

Hard-working students covet trophy by nature. Not winning may wound them psychologically and leave them wondering where it went wrong or if they’ve done a lousy job of it. Transparency in the marking schemes would do much to explain as well as help them prepare. I would like to let them know the criteria.

Dr ******

Much like what they do in shows such as Idol, the panel and the audience may well be exerting equal weightage. We now have the verdict of the judges only, and the panel is made up of only six. We could use another consideration for an overall conclusion, i.e. taking soundings in the form of the participants’ votes.

Dr ******

Last year our favorite group went home empty-handed. Not of our department, but very composed. Excellent presentation, we think. Yet not a trophy to their name.

Dr ******

A shame that we did not win as expected.

Dr ******

The perception gap is down to the speciality difference, to be honest. Wide-ranging subject matter at a conference that is evaluated by, say, pathologists for researches presented at Pathology Society. The same goes for the DMR-organized research. The best almost never finishes first. One reason for this is a lack of an in-depth understanding of the topic. The prizes are scooped by the best presenters. Few are the judges possessing a specific knowledge of the topic, that’s probably why.

Dr ******

There must be specialist judges. And external judges, too, to be fair and square. Were I on the panel, I would be of a biased opinion leaning towards presentations with a focus on my speciality since that’d be featuring a lot of details I’m able to appreciate. Less so in case of other disciplines. To give equal chance, two judges of each subject would be okay. One more judge with a certain expertise might tip the scales towards that particular field of study. Naturally the parent of a presenter shouldn’t be placed on the panel.

Dr ******

Students doing everything shouldn’t be the way to go in RCA. It sure is a delight to watch, these twenty or so students in uniform all fired up. Still the university could be doing its part, I think. Now the financing, the invitations, the event-planning and reception are all on them. The other events are usually organized with the help of the Admin and Estates offices, but in case of RCA their services are limited to allowing hall access, seating and microphone setup. The students being unable to deal with contingencies, any disruption to the event plan can cause a commotion.

Dr ******

While we’re on the subject of judging, we junior teachers don’t know how to respond to a group enquiring why they didn’t win. So feedback on each group might help them realize the respective shortcomings that cost them the prize.

Dr ****** Dr ******

Dr ******

The prize of 50 thousand kyats to be shared by a 40-member group won’t amount to much. Not even for a treat. And which one of them is to keep the certificate as a memento, they argue. The hardest workers in the group would claim the right. This is resolved by issuing copies. It’s hard to know for sure how much of a roll sheet are included in a given group. The presenter most likely wins the privilege in the end, and the data collectors and other members would have nothing to show for their accolade.

The End.

**Student FGD1**

Q: Knowledge, attitude and skills gained from RCA

Student *****

We can find out thanks to such a research whether the theory we’re taught tallies with what is in practice at the hospital. It also highlights the differences if any. Our group’s lot is ‘drug utilization on peptic ulcer’ investigating if or where the drug use at the ward differs from the textbook regimes.

Student *****

We studied antibiotic susceptibility for our pharmacology research, focusing on the reasons behind drug resistance of tuberculosis. We were taught at uni causes like inadequate duration and not enough dosage. We meant to find out if that is the case at the hospital. We personally collected the case details of over 400 patients. All the data on antibiotic (anti-TB) use. By jotting down the specifics we got to learn new names and google them for more information. Some solid knowledge amassed that away.

Student *****

Taking on microbiology, we tested for antibiotic susceptibility. In the past coagulase-positive staphylococci were more antibiotic-resistant. Resistance is now found to be emerging in coagulase-negative strains of staphylococci, alerting us to the proper use of antibiotics.

Student *****

I crunched the data for the ‘antibiotic resistance’ group of pharmacology. I had imagined this as a task involving the differentiates or integrates and the cushion curves. As it turned out, it was not so difficult and was limited to the calculations of mode, frequency or quantity. Lots of data on one drug, computed for both genders. What I learnt at the time was that the frequency, the mean and the mode were in widespread use, not the high-flown stuff as anticipated. To my surprise these seemingly insignificant tools of calculation we learnt as children were really handy!

Q : Attitude towards RCA

Student *****

I’m starting to see how important research can be. I did not think so back then as I was far away from it and was research-naïve. In order to produce concrete results, a jack of all trades like myself needs to be precise. One research’s conclusion is a basis for further researches to build on, as is the way with evidence-based medicine. Like, which microbes are susceptible to which drug, when it comes to antibiotic resistance. We administer broad-spectrum antibiotics when these are not necessarily prescribed in other countries. Drug resistance rears its head when research is not as commonplace. Now we have a chance to develop skills including precision and teamwork. This cannot be accomplished by one person alone. It’s a collaborative effort. And a race against time, considering the 2-3 week countdown.

Student *****

What is apparent to me is the difference in individual skills, for instance computing or using Excel. With regard to knowledge, we were observing the culturing of microorganisms and none of us was able to answer well when asked by our paediatrics teacher at 550 what the common bacteria in children are. Could not exactly tell which organism was gram-positive and which was gram-negative. We’ve had our study, but not exposure. It’s a relatively new subject for us, so we didn’t do well with all the questions shot by our teacher. She did teach us properly. Seeing someone a generation older than us so active and energetic, we could not afford to be slacking. We went to her in pairs and heard only good things from her. That was uplifting naturally. No shirking, since even our great teacher can be seen still toiling away. We formed teams according to expertise. The girls were to read up on the subject, respond to the questions and write literature review. The boys must take care of all technological aspects. Most of us wouldn’t complain. Of course there’s one in every group, but 80% were very active, as I saw it. The research had brought us together as a team. And the knowledge acquired is great but hard to retain. Research was not part of the final years’ curriculum. It will surely come as something new when we deal with it doing a master degree. All in all, what we’ve really gained is teamwork, plus the spirit and leadership of the teacher.

Student *****

I can only give a bystander’s opinion; I wasn’t a part of RCA, being involved in another event at the time. Working in teams does much to get to know one another, and labour is distributed according to skillsets. As KSH has said, all persons involved are only trying to make this one-time event a success.

Student *****

Why not do this every year then so we’re no stranger to research culture?

All:

Research culture should be introduced early in the medical studies. The thing is we have time management issues, juggling RCA poorly with clashing exams and ward postings.

Student *****

Research may ideally start in high school. Unfeasible as it is, we experience this in our university life; it could start from Second MB onwards though.

Suggestions towards RCA

Student *****

Research should be introduced at the foundation year, I suppose. Designing systematic coursework that includes step-by-step basic patterns aimed at the overall skill persistence. The key is impressing upon the students the purpose of studying something, only through which students may persevere of their own accord. Another point is choosing research areas. These must be of interest to students; preferably the hot topics. Prioritizing these areas of interest drives home the message that the medical field is evolving and our knowledge needs continuous upgrading.

Student *****

I’d like to report the difficulties encountered. Our group of 41 students is responsible for poster presentation. All 41 except one or two were involved in the data collection process, but once the data were in hand, many were sitting out and needed a lot of pushing. Data collection entails only noting the case information, that’s why. Only eight of us were left to handle the rest that followed, and we were swamped, with all the other classwork. Consequently our poster contained errors, even after five times of editing. Still the final draft wasn’t error-free. An eight-person workforce could accomplish only so much. These flaws would have been rectified by half, if not all, of the 41 members. It was no wonder really we failed to clinch a prize, competing with weaknesses. We aimed for the first prize but had to settle for the result produced by eight persons’ worth of efforts. I’d like to state here that a leader shouldn’t have to do everything and individual members must have a sense of responsibility and leadership. I was not a leader but saw what I could do for the leader and the group’s welfare. With all the classwork in hand, I strove all day while some of the others wouldn’t buckle under the calls for help. My point is every one of us ought to have a leadership mentality and team spirit in order to overcome the challenges daunting as they may be.

Q:

Student *****

I would prefer RCA to be tied to classwork. That way we can make things work even if three out of the 41 members are missing. All the members should see the process through to the end.

Student *****

I’d like to offer my suggestion on the issue of teamwork. I must say our supervisor excels in management. We were put into small groups of four, each with a subleader. One team digitized the data gathered; one containing smart students took care of the literature. Another team was set preparing the slide presentation, and there was a team reserved for the miscellany of tasks coming up. The supervisor had considered the respective skills of the members when forming teams, so it went very well and we won the first prize. It was all due to the brilliant organizational skills of our supervisor. Indeed supervisors need to be strategic, laying down frameworks for the research. A variety of frameworks to choose from. If such frameworks were set by the university, 80% if not 100 of the overwhelming workload problem could be resolved. The roles would otherwise be ill-defined. But for the teacher defining these roles, it would’ve ended up very messy, with our strained relationships. The specific team roles also made for effective communication.

Student *****

As my friend has mentioned, we were an eight-person task force, not 41. The same way with most groups, even carefully structured ones like MST’s. I asked my classmates this: “do you agree to take me as your leader?” And “who will be the presenter?” We volunteered rather than take a vote. I’m of the opinion that all of us eligible for medical universities have the same level of intelligence, IQ or EQ, and that there’s a leader in us all, suppressed by not claiming a lead role, and having someone else lead right from the first year makes that person indispensable. Our group, with all the data crunching, entered the preparation phase only one week ahead of the event. The role of presenter had not been taken. Not by vote. Someone capable, not letting his skills go to waste, would be there to assume the mantle of lead presenter. If a group has a designated presenter, only he or she will be able to do the job. So we didn’t use force; instead we showed them appreciation. As for members with no special skills, we let them print out the poster; for their effort in printing they had played a part, and there was no one with zero effort. I wanted all the members to perform their favourite tasks. One of them finally got back to me saying how he had committed the whole poster to memory and wanted to present. I trusted him with the role he himself claimed. No need for additional pressure. He did his best in presentation. We must also reach out with mutual respect to the less active members rather than dismiss them as good-for-nothing. Relationships cannot be strained, or dominated, since these are the guys we’ll be working with throughout the part-1 trip.

Q: Difficulties

Student *****

The problem with our data collection is the switch in hospitals. We’re supposed to monitor the selected cases for antibiotic regimens in the three weeks given. But with the transition between wards, the data of some cases were not complete. Moving on to the 300-bedded Hospital after one month at MUIII, we were hardly able to squeeze the data collection at MUIII into our lunch routine without skipping the afternoon session at 300-bedded, hence the case collection at the latter. Our supervisor would rather we had studied at the same site, considering also that there’re not as many cases at 300-bedded and each of us were to take ten cases. We prefer, if at all possible, staying on at the same ward while conducting research. It was not just us; all the groups had to go through the ward switch. Since we have a month’s stay at one ward, I propose that RCA should be timed to coincide with the start of a ward posting.

The data collection went off without a hitch. Almost all the members did their parts. Don’t know about the data reliability, though. Some may not have visited the ward daily. Some copied the regimens straight from the charts, and the complete data on the drug use for the three weeks were not recorded in some instances. We had no way of knowing for sure whether or not each member had observed their share of cases throughout the entirety of three weeks. There were leaders keeping tabs, but not all the time. That means everyone should have a sense of responsibility that needs no urging on. Consciously fetching the data every day after the lectures. I did miss a day or two but went there about twenty times. Some cases were discharged, and there was not much change in the courses prescribed to the remaining in-patients. We only needed to check. Conscientiousness was all that’s required.

Student *****

We only needed to prepare a paper and a powerpoint from the data collected by the teachers. We were unique in that we didn’t have to get the data ourselves. This our professor noticed, too. Personally I would’ve preferred gathering the data for the sake of teamwork mostly. Now that this part of the job wasn’t ours, we only had four or five working members. Not participating denied our members the insights, and the experience of observing sample collection and culturing shown by our teachers. They only examined the ready-made slides under a microscope.

Q : Why?

Student *****

The teachers chose to do this. But for the coming years, my wish is that our teachers would have students collect the data on their own, just like other groups.

Student *****

I didn’t go there every day. Only a couple of days, since I could refer to the charts containing the whole month’s worth of data, even those of the discharged cases. Of these charts, the ones detailing antibiotic use were specifically analysed. It took all day and was exhausting, but it kind of solved the issue of us switching wards. Since we didn’t have to do it every day, we could stick to one hospital. Not a problem with one or two days of data collection there. The teacher at the ward would ask and teach us salient points whenever we visited. We learnt first-hand the culturing of many microorganisms as well.

Q: Difficulties

Student *****

Normally we students were only on nodding terms with the teachers. But in the course of RCA, we interacted with them on many occasions, via messenger groups or phone calls, and this deepened our ties, as with the integrated teaching in the second year.

Student *****

We got on quite well with the supervising teachers. The problem is with our classmates: about two out of a six-person team cannot be made to participate, with the result that when met at the hospital they came to be viewed not so fondly but as slackers. As we age we become more conscious of equitably sharing workloads, which when overwhelming can really test our tolerance! This lack of participation may be addressed by stressing the importance of an assignment enough to convince each student to devote. Of course this would be left to our teachers. Any student doing so will only be written off as domineering or something.

Student *****

A group of 41 students is too large, I think, to be managed and overseen by two supervisors. Two subgroups could work on a topic and compare notes to spot errors.

Student *****

I’d like to make a suggestion concerning data. I believe there should have been two or three days of training in advance. As someone who has been working with data for a living, I question the authenticity of the data generated. The students have provided the data in quantity, but what about in quality? Our group managed to capture a wealth of data. But one of the other groups that presented a smaller number of data somehow made it work and won, not that we’re disgruntled or anything. Just doubting the quality of it all. So I want us to receive a crash course on data. A couple of days’ training won’t make professionals, I know, but the insights and exposure alone can do a lot for result accuracy. I expected extensive coverage from the orientation course. It did touch upon the data handling, yes, but the focus was on research methodology. We should’ve devoted a whole day to data. The instructor shouldn’t be insistent on Microsoft Excel only out of expediency when a multitude of workarounds may also be shown; it’s not like we doctors are doing this professionally. I suggest we start with the different types of data and how to collect them, then working our way up. Not to be expert but just enough to grasp the essence. This will give us a whole new perspective on the process of data collection. So what I would love is a sound theoretical basis of working the data, including the types and models. Essentially a brief recap of statistics. Only after such a course will we be delivering more accurate results, I expect.

Student *****

According to our supervisor, we could ask the nurses or doctors what antibiotics are in use and what the ampoules look like, and they’d be willing to help. When we did go and ask what a drug’s like, no one was there to help. Only the charts from which we copied. A communication problem exists between the hospital and students. As for the communication among us students, I was the designated techie, albeit not particularly adept at using Excel, and I had to do my part in the group coping with an abundance of setbacks. Probably down to the lack of expertise, but we had a hard time fitting over 300 papers in a table and our draft needed editing many times over, even using the department computer sometimes. The teacher helped us, she did. But through our lack of skills the process took up an inordinate amount of time. We asked around, and the others were equally incapable. So an Excel proficiency is needed. But no such training. Meaning students go about this as best they can, under some guidance that is. Further, some students simply dropped a shot of, say, acknowledgements in my inbox and were done with it. We the typists were the ones showing it to the supervisor and getting a no. We wouldn’t know how to redo it. It’s their share of responsibility, not ours, and delay’s unavoidable when they neglected to participate. Such was the communication problem. We tried phoning at the supervisor’s behest but there was no answer.

Q :

Student *****

The hospital on their part should have a person charged with student affairs. That teacher could then show and explain to us common antibiotics so we may photographically record the packaging of three or so vial preparations and refrain from using googled materials.

Q :

Individually they must come; most didn’t find the time. We had to carry this out expeditiously at the last minute. The supervisor should’ve called us separately, one team after another. For instance, the acknowledgements team, only after working with our teacher, could’ve handed the finished product over to us computer guys. In that case we only have the typing after a few corrections of mechanical nature, much to our convenience. What I mean is they take complete charge of their end as ours is limited to eliminating typos. We’d be more than happy to receive the final version that has been run past the supervising teachers.

Q : Difficulties

Student *****

A presenter needs to be involved in every single facet, I think.

Student *****

The one on the receiving end of all the questions, the presenter certainly needs to be immersed in the whole process. He or she has to invest the most time, attending to the data processing passively at least.

Student *****

Our inadequacy in computer skills posed a problem to preparations. To enhance visibility in the Excel cells, we entered short forms like AA or BB for drug names. I know about sheet files and row files, but no time to explain really. Each of the group members, with no prior training, took turns daily to code and read out the names plus the equivalent letters to enter. The good thing about our group is everyone was there, or at least playing games nearby ready to take over when the working classmate needed to catch their breath. We had a pretty tight group throughout the preparation, especially the tech guys. Microsoft Excel presented great problems. Some need for training there. The teacher wasn’t helping. She had entrusted everything to one senior student who was the mainstay of the group. And we were overwhelmed when he was absent. He came when Excel stumped us. Still the group was clueless about what to do. It was not as if the two teachers could feed the info and updates to all forty of us individually. We assembled for this purpose but there were no-shows. And those absentees in their own sweet time did finally come, but there were no specific tasks set after the breakdown of the info link. Too many can also be a problem; we had a score of redundant members. That much manpower available for reading, data collection and writing acknowledgements. We have enough hands in preparation. And what’s more, things will be made easier with preset training.

Q : presenter’s difficulty

Student *****

Can’t say I’m content with all the powerpoints prepared. If not self-prepared, they most likely include whole paragraphs plagiarized. I failed to connect during paper preparation with the powerpoint-maker. I should’ve been beside that person revising and amending certain aspects. I see now three or four groups featuring cut and pasted paragraphs recited by their presenter. No bullet points in these paper presentations. Nothing of the like seen in posters, though. On another note, the presenter has got a lot to read up on, like foreign papers from India even. We’re pressed for time, with a lot to read and a deadline to meet. The teachers recommended the journals to study, though.

Student *****

Well, the research title should be given at the start of the academic year. The projected area of study to choose a title from would be fine. That’s for papers. Posters are not as difficult and will be okay.

Student *****

But even posters some didn’t read. They were like ‘vibrant colours there, mate’ at the final draft. They seemed to have shut down after their part was over. The presenter had to do everything in our group. There were some who, only after we’d won, came to read the poster. Everyone should’ve read at least once. The teacher did the urging, explaining how it’s exhausting and the presenter shouldn’t be the only one taking the questions. Indeed the high-achieving students and any member with time to spare should be studying for the discussion. In our group three of us had been chosen to respond to the questions, but the presenter ended up answering all. So if every member has read the poster at least once, they’ll know enough about the work to answer some questions shot.

Q :

Student *****

Communication was smooth. But so many changes and modifications.

Student *****

The supervisors, as the term suggests, only laid down instructions. They gave a title and said the word go. Not much beyond that. They disliked this part and that but wouldn’t elaborate on how to make it better. They could’ve been more helpful, given that it was our first experience.

Student *****

That our supervisor was. Helpful, down to the last detail.

Student *****

It’ll be his/her paper in that case!

Student *****

We edited the reference section around ten times. In minute detail that was, font, punctuation and style of citing the journal titles. We had to search for these journals to refer, and that took time. One merit is unerring accuracy, though the details could’ve been intimated beforehand. Giving us guidelines and formats ahead of the task would save the trouble of the all-too-frequent amendments.

Student *****

It so happened sometimes that we noticed mistakes after printing our the poster. What’s done was done; we couldn’t change the poster anymore. So we just corrected what we showed on the projector.

Q : other suggestion ?

Student *****

The benefits I got out of research include the leadership skills and potential. I was not the leader. But our leader had been EC and I learnt a lot from him. He’s got an extensive knowledge base. When our teacher didn’t give us a to-read list, he downloaded some journals for us to read and showed us how. When he and I distributed work, he took intro, methodology and objective sections without exactly specifying who’s to do what. I’m not good at management, just starting out. Not even aware of the contents. So we got through this by improvising. Here the supervisor should’ve managed, yes, and set duties.

Student *****

The supervisors had us hand in our works too late and close to the event to make necessary corrections. That was inconvenient. We could’ve set an earlier date and taken our time improving on the work.

Student *****

We went there three times a week for data collection. The consultant in charge there at GI Ward explained drug regimes in PU as well as case selection for *H. pylori* eradication. She took us on rounds and let us check the charts for drugs used. On occasions we had to interact with the patients, for example to see the clarithromycin one patient was taking, and it went well enough.

Q: suggestion

Student *****

I don’t think our uni has a format style for this. We did refer to an award-winning poster as a template. But we didn’t know to go into detail at references. Then the supervisor showed us a sheet of paper with different referencing styles on it and chose one that suits the uni. Not sure our uni has an official style. The styles varied from journals to papers, I found. In fact a format preset by the uni would be better.

Student *****

There was a lecture on formats, but not before the research. Shouldn’t it be delivered in advance?

Student *****

Yes, there was a knowledge-sharing session on research long after the activity.

Other suggestion

Student *****

I would like the winning papers to be published – first, second, third and consolation. Then all the students, not just third-MB, will have access to the papers. Pre-training is another point: our Student Union did invite a freelance teacher to give a talk on methodology but falling on a Sunday this open event failed to captivate many students. I want to suggest, if possible, planning many training sessions in the run-up to RCA.

Student *****

You can count on the elite students to be absent at weekends. No way they’re cancelling the tuition classes.

Student *****

They see no tangible benefits. Might as well use classwork as a tool, or like that time the attendance to get them in line. And training should be conducted in groups no larger than 30 or 40-member, much like the ward training. Social Media Ethics has once been taught at the foundation-year level by our forensics teachers. The use of focus groups, as I see it, was the reason behind the success.

**Student FGD II**

Student *****

We at pathology studied sexually transmitted infections. We had to visit STD Clinic, which was not part of our third-year ward posting. The STD Clinic gave us the experience of exercising caution in dealing with these cases. We must be careful what we say to STD patients and respect their privacy as well.

Student *****

Many cases there. I saw IVDUs and cases of syphilis, sometimes couples. Some were very young. The drug users were there every week for their prescriptions, no tip-offs. They trusted us enough to confide. Told us they’d just started using. We got exposure to these cases, and they were polite enough.

Student *****

We investigated the seroprevalence of Hep B, Hep C and HIV among the blood donors at MGH. We acquired consent. Had them read the consent form, explained and requested interested individuals to participate. The time frame was one month, with Test 3 in the way. We got over 200 samples in the three days pre-test and went on collecting thereafter, with donors’ consent of course. We didn’t note the names, only the date and other info. Not being able to identify the exact cases when we were back for results, we retrieved the data just like that; so we did give time for consent but the cases collected may have included those with no prior consent. Afterwards we worked the data and got zeroes for a few parameters. The 200 samples didn’t include one positive for certain specifications. The sample size was made larger accordingly and consent was no longer a must for us this close to the exam. The nurses were requested to take it but everyone helped. The donors mostly come in groups; we only need explain to the leader, a venerable monk mostly. We got loads of experience in social dealings. No one really paid attention when we did the explaining for consent. And no one volunteered first, as far as I know; all believed they were negative for B, C and HIV. They accepted when we offered to contact them in case of positive test results. They saw this as a perk.

Student *****

We did research on microbiology. No direct contact with patients. We only collected results. For that we went to the microbiology department of 550-bedded Children’s Hospital on 66^th^ St. The teacher there explained the procedures including the culturing and sample collection from blood, urine and ET swabs. Depending on the clinical specimens it takes 24 hours to one week. When the culture came back positive, drug susceptibility was tested and the data on positivity and negativity for different drugs were collected. Our group consisted of thirty members. We went there in pairs each time, every three days since the results were not instant. As the exam drew nearer, time management became an issue. All of us had to work on the data a lot in the end. Our teacher on her part taught us methodically how to culture in addition to other practical aspects of microbiology. Some of us asked the teacher what they didn’t understand. Some just collected data in their short lunchtime.

Student *****

It was our teachers that selected the research area in the preliminary phase, a title that they liked and that worked for us. There were thirty of us. Not everyone was participating, but fearing a falling-out we were not on their case. Also, we had the morning lecture and the afternoon session at the hospital, with only an hour of lunch break in between. Not skipping lunch or wanting to be late, we ended up cancelling the afternoon practicals and used the time for the research work.

Student *****

We had to request leaves of absence at the hospital.

Student *****

We subdivided our group, half of us attending practicals at the ward and the other half going to the STD Clinic. Both the leaders, as it happened, went along on data collection, and the rest of us at the ward had to ask the teachers for their leave. Some teachers were not as willing to give them a pass. We had some attendance issues.

Student *****

Our group contained members of two different ward postings. Noticing low attendance at the wards, some teachers refused to compromise without the rector’s signature, which we had to secure. We don’t know about our attendance, but we did provide them with the signature. It’ll be favourable for the next batch if the official paperwork precedes them.

Student *****

We collected ovarian tumour data at Onco Ward. History taking was short; we focused on the ultrasound findings, to be shown on the projector to our teachers for differentiation between benign and malignant nature. But only the leaders and the presenter were actively involved. Some of the rest were absent or late. They only cared about their roll call. No attendance collection meant no appearance. With only the teacher and a handful of students we ran into some difficulties.

Likewise!

There were so many among us not lifting a finger to help.

Student *****

The supposed all-inclusiveness did not include all.

Student *****

Better if each group’s dispatched with a teacher leading the charge. And collecting attendance. Students go alone now and our teachers are only there for introductions. We want our teachers with us every time so the hospital side respect this.

Student *****

Even if we students take attendance, it’s not effective since our friends can simply phone us for a favour, which we can’t ignore.

Student *****

The workers worked, and the shirkers shirked.

Student *****

Pretty much the same here, run by only four or five working members.

Student *****

We studied children hospitalized with renal oedema. They’re in and out of hospital staying month-long each time, hence the issue with our sample size; see, the forty of us were tasked with collecting three cases each, a total of 120 samples. Not nearly as many inpatients there, so to fill our quotas some of us including myself ended up case-hunting at OPD. The OPD team led by me had to work through many different cases, most having a long history of hospitalizations and steroid treatment. We leafed through the records scanning for required information and taking photos in the time we had. We tried locating the data like duration, blood works and the presence of proteinuria. Not so convenient with outpatients. We got only around 50 cases.

Student *****

Very common problem, yes.

Student *****

As for us, 200 was projected and approximately 1,000 samples were collected.

Student *****

Cases were rare. Same patients every week.

Student *****

We collected STD data. No confusion between new and follow-up cases. The sample size wasn’t met before we mass-collected the cases. At first we only recorded case data from Jan 2019 onwards, many of which being incomplete had to be excluded. The sample size was filled by history-taking directly from the patients attending the clinic, 200 projected but roughly 90 collected. We had to make it work.

Student *****

The data recorded were not complete. Scant information on gonorrhoea cases. And only one or two symptoms were elicited on history. The diagnoses were not conclusive. We found, upon inquiring of the nurses, these were more conjectural than evidence-based. Apparently certain presenting features according to clinical experience call for certain treatments. The best they could do, they explained, under overwhelming caseloads.

Student *****

Suppose we managed to capture a few cases seeking medications this week but we needed more data on them; they’re not there the following week, and the records kept there were not helpful either.

Student *****

We collected more than 1,000 samples’ worth of data over several days – unthinkable really to get them all in one go. We copied the data anyway and worked the percentage for the collective positivity. We were in a tight corner, with the timeline moved up and pushed back. And the clashes with ward posting became a common excuse among the classmates.

Student *****

An extended period will play this out comfortably.

Student *****

Research alone will be fine. Not doing well at all with the hospital, the lectures and the research.

Student *****

There’s also the tutorial after a lecture. Then we can turn to research, but not unless the tuto’s dismissed early enough.

Student *****

Regarding the topic choice, enquiries should be made beforehand whether our teachers’ research interests will be supported by the sample inpatients. That was not the case in our group. Connections were only through a demonstrator and her paediatrician friend who asked the higher-ups about feasibility, not directly through the authoritative counterparts. The scarcity of case data was the result when we actually went collecting samples.

Student *****

The title should’ve been considered well ahead of time, especially how the case data collection would go and if there’d be enough literature to back it up when students present. Now the topics are chosen on the verge of research activity and changed when they’re not working.

Student *****

Yes, that’s also how it was with our groups.

Student *****

They should’ve tested the waters.

Student *****

The junior supervisors were all right. And pretty solid, too.

Student *****

The poster dimensions should’ve been communicated in the first place. They turned out different from department to department and had to be made consistent among us. The fonts, too. The font size should’ve been the same from the start. The printings cost us a little more now.

Student *****

The amount we had to spend was 5K per capita, making 150-200K kyats for a group of 30-plus students. Worse still, we published only one day ahead of time; the printer’s wasn’t prepared to handle the workload this close to deadline and we had to force their hand. And the teachers revised a lot, some up to seven times! The whole activity’s disjointed: led by one party, prepared by another and printed by yet another. Our five leaders were engaged in the event-planning and could not turn to the publishing. Some members stepped up, thankfully. The thing is the final printing’s possible only with the works of all three departments – the books wouldn’t come out if one group’s running late. Three-day countdown, and printing’s only beginning. One week will suffice well to prepare the books.

Student *****

The matter of funding should also be reconsidered. Shouldn’t just fall to us; the uni could be providing if only half. We could only afford our teachers’ refreshments, and if not sponsored, we’d have had no financial means to be catering for our studentfolk. We had collected 5,000 kyats from each; we didn’t want to serve them bread, so we collected more. But less presentable were the homage-paying gifts, each teacher only receiving a bath towel.

Student *****

We were aided financially.

Student *****

One suggestion here is we set a date and a person for printing. Now the other groups had to wait when one group’s not done with revising, and that delayed the printing process. The poster vinyl took one night minimum and needed checking for ink smudges the next day. Why not reserve these three or four days prior to the event for preparation, might I suggest? And the number of copies needed vary: we set aside a certain number for judges and for the APs, some departments getting more books and some not getting enough.

Student *****

There sure were difficulties. Our group distributed labour to streamline the process. Still no amount of action planning could keep some of us from dodging work; it’s a point of contention. We’re on good terms with our leading teachers but not quite with the clinic. They’re busy most of the time at the clinic, so. The supervisor accompanied us for introductions on day one and gave us orientation. They treated us well on that day, but later when we were alone, they didn’t show us much respect, not even the medical officers there. We abided by their rules but they didn’t appreciate us inadvertently overstepping sometimes, like when we took history and some questions were deemed intrusive.

Student *****

Next up is supervisor-student communication. Amid all our lectures and tutorials we managed to collect the data, but we had only the lunchtime to present these to the teachers. We worked at weekends too, when our teacher was otherwise engaged; so we made contact by phone or Messenger. What we’d prefer is to make a rendezvous at Library or somewhere on days off for instructions, if they’re not free on weekdays. Now Messenger’s the place; we submitted something and kept revising the parts they didn’t like, the corrected drafts of which were shown to our senior supervisor. She’s mostly busy, they kept communicating on Messenger, and that held things up. Inordinately time-consuming, it was.

Student *****

As for the first day of our data collection, the supervisor only phoned the ward, which wouldn’t let us in. Took quite a bit of persuasion, and our dean’s signature. They were not apprised of our research. So not so accepting of the sudden large number of students before the paperwork. In the future the ward should be properly notified of the incoming students by one of our teachers.

Student *****

There’re upsides to certain aspects. The working members were gaining momentum on what they’re doing. We had a couple of members who read and read providing highlights, so the task of writing fell to some other members. Some who had distanced themselves from the work were getting more and more distant. So much so that the non-working bunch didn’t even attend the event. The questions were taken only by the members exposed to the work. When they couldn’t answer then no one else could. And gone were the speaking terms, post-RCA.

Student *****

We at microbiology consolidated a sound theoretical base. But not practical since we only collected the data. Some of us were no longer Excel-naïve. Some cultivated a reading habit. Whatever it was we were doing we improved, while the form of our inactive members dipped. They did offer to help where we needed them, but we wouldn’t waste our breath. Take, for instance, when we met on Saturdays: our more driven friends arrived early and would not wait around for their tardy classmates. But these classmates did help us out with photocopying and procuring books at least.

Student *****

We developed teamwork. Of our thirty members, five were very active, 15 were neutral and the remaining ten were apathetic. We knew their types and paired them up accordingly. We defined our respective tasks: printing, computing, for some girls data check and cleansing, and presentation and literature study for five or so members poised to take questions. No member was left to idle.

Student *****

I suppose we have learnt a method or developed a pattern for research work, including data collection, drawing charts, percentage calculations, etc. We also enjoyed the reading exposure to our reference journals. Our teacher provided us with these journals, which we distributed among ourselves.

Student *****

We were exposed to research, something we had only heard of. We now know the contents, what follows the introduction and such.

Student *****

There was a session on research organized by a teacher. A short course on paper and poster preparation. This has conveniently familiarized us with the procedure, like what the abstract ought to cover.

Student *****

That was after the RCA.

Yes, preferably before.

I gained more from self-study. I was asked to draw charts and tables, which I didn’t know how to do. I googled and studied the steps. And the responsibility. We each must take this seriously or we start from scratch when one of us messes up somewhere. It teaches us responsibility.

Student *****

I suggest the leaders should be called up earlier for event-planning. They’re singled out, as it were, as close as one week to the event.

Student *****

Each group has got a leader. The meeting for planning the event was called only one week before, only at which time the dates for printing and such were discussed and set. The schedule was pretty tight. They put up the banner only in the morning of the event. And it took a lot of pleading with the caterers, who had difficulty meeting the deadline and weren’t keen on working at 4 am.

Student *****

Our group leader was also an event-planning leader and could not make time for the research. So we had to recruit the help of someone else for overseeing the powerpoint preparation. The event-planning took all day and included logistics. We were short of manpower and time. It’s inconvenient for a student to double as team leader and event-planner.

Student *****

We studied STIs. We knew they were prevalent but had no idea they’re that common. So many cases there. In order to reduce the incidence, the public should be receiving more health education. It’s not just illiterates that are afflicted, graduates too.

Student *****

For motivation of the next batch, I’d prefer trophies to certificates. Only one certificate was presented to each group. Instead trophies could be showcased or displayed for better effect.

Student *****

The certificates were printed only one day ahead. Reprinted three or four times. The leaders were considering giving trophies. Our budget wouldn’t allow us.

Student *****

While we are on the matter of budgets, it would’ve been more convenient to project an amount to collect, taking into account the printing, event-planning and the catering. When it happened that we were short, raising funds the second time around didn’t cover some students. That was inequitable.

Student *****

It’d be wise to get a rough estimate of the amount spent by the last batch. Then the leaders and the teachers may discuss this and raise the projected amount before the research. It shouldn’t matter much to err on the side of more funds; 10K is better than 5K twice as some may be going on about how they’ve paid already. Collecting money from the whole class with bowls in hand isn’t the best way either. It’ll work well for the group leaders to collect their respective lots.

Student *****

Students before pursuing research work should reach out to the seniors who can recapitulate on the whole process because it isn’t ideal to be learning the ropes as we go along. We should’ve arranged a meet-up to gain some insights.

Student *****

This is a school activity, the way I see it, related to all three departments. They should’ve proposed a budget, if only half, to the university if they have this RCA in mind. Even 500K or 100K from the uni should relieve our students’ burden as each event costs 1.5 to 2M kyats. On to the matter of catering, we have three parties in teachers, students and admin staff – teachers supervising their students’ research; the admins should be doing their part to take care of the event-planning.

Student *****

We studied at paediatrics the gentamicin or piperacillin resistance of microorganisms from blood, urine and ET tube samples. We searched for drug information, not the journals though.

Student *****

Our group searched for journals. The teacher gave us five, but we searched for more when we got the data. One thing inconvenient at the event was we were asked in relation to what. Not Myanmar data. And it occurred to us that we should also be looking for local data, at the library maybe. We couldn’t find many accessible, only two or so. And these two focused on Hep B or C separately.

Student *****

Not at the Third-MB level, no. Perhaps not even at Final Part-1. It wouldn’t be so bad if Part-2 or internship were to tie in with a compulsory research culture activity. But not Third-MB. I remember a friend of mine suggesting a study on hypertension, the research area he could think of, and another on diabetes mellitus and blood sugar levels. Neither of them could come up with a specific viable plan. So it’s appropriate that topics are set the way they are at this third-year level.

Student *****

I’d like the winning group to be awarded a trophy and each member to receive a certificate with the name written on it. Now one certificate is given to the whole group from, say, roll number 100 to 150. Everyone wants it, but who is keeping it? They want something like this research on their career records. It’ll be great if certificates are awarded to all members and trophies to the three winning groups.
