# Supplementary material for: Perceptions of faculty and medical students regarding an undergraduate research culture activity in Myanmar: a qualitative study
Source: J Educ Eval Health Prof. 2025 Oct 27;22:33. doi: 10.3352/jeehp.2025.22.33 (PMC12768548; doi:10.3352/jeehp.2025.22.33)
Supplement: Supplementary file 2 — Supplement 1. RCA program snapshot (UMM, 2019–2020). [file jeehp-22-33-suppl1.docx]

**Supplement 1. RCA program snapshot (UMM)**

| 1. **Curricular status & level:** Compulsory, cohort-wide activity in the third-year para-clinical phase (Pathology, Pharmacology, Microbiology). 2. **Duration & timing:** Scheduled as a two-month block (November–December) within the academic timetable; presentation in early January. 3. **Cohort size:** Approximately 300–400 students (varies by cohort). 4. **Grouping:** 30–35 students per group; typically 10–12 groups across the cohort. 5. **Supervisor assignment:** Students are allocated to one of the three para-clinical departments by roll number; groups are supervised by departmental faculty (from demonstrator to associate professor ranks). 6. **Topics & designs:** Topics are selected or assigned within departmental scope; projects emphasize feasible designs, commonly retrospective or cross-sectional. 7. **How students and supervisors worked together:** Regular team meetings and iterative document revisions; basic data handling/analysis (often spreadsheets); formative feedback cycles with supervisors. 8. **Support:** limited and informal (logistical/research-technical/software-technical) support provided within departments guided by the assigned supervisors; 9. **Outputs & reporting:** Each group produces a poster or short paper and presents at the RCA day with formative Q&A/feedback. 10. **Evaluation:** Emphasis on formative feedback; any summative use of marks varied by department and was not systematically studied in this project. |
| --- |
