# Supplementary material for: Perceptions of faculty and medical students regarding an undergraduate research culture activity in Myanmar: a qualitative study
Source: J Educ Eval Health Prof. 2025 Oct 27;22:33. doi: 10.3352/jeehp.2025.22.33 (PMC12768548; doi:10.3352/jeehp.2025.22.33)
Supplement: Supplementary file 3 — Supplement 2. Interview guide for student and faculty FGDs. [file jeehp-22-33-suppl2.docx]

**Supplement 2. Semi-Structured Focus Group Discussion (FGD) Questions
Used to Explore Experiences with Research Culture Activity (RCA)**

| Questions of FGD | I. Which kind of “Knowledge, Attitude and Skills” you have gained from research culture day activity (RCA)?*  II. What kind of challenges that you had to encounter during RCA?  III. How did you overcome the challenges that you had to encounter during RCA?  IV. What would be your constructive feedback, if present, regarding RCA for the betterment of curriculum planning in the following years? |
| --- | --- |
| * I. is to ask for Student FGDs only. And II., III. And IV. are to ask for both Faculty FGDs and Student FGDs. | |
