# Supplementary material for: Perceptions of faculty and medical students regarding an undergraduate research culture activity in Myanmar: a qualitative study
Source: J Educ Eval Health Prof. 2025 Oct 27;22:33. doi: 10.3352/jeehp.2025.22.33 (PMC12768548; doi:10.3352/jeehp.2025.22.33)
Supplement: Supplementary file 4 — Supplement 3. Illustrative quotations (Theme 1–12). [file jeehp-22-33-suppl3.docx]

# Supplement 3. Illustrative quotations by theme (Themes 1–12)

This supplement presents illustrative verbatim quotations from students and faculty participants, organized by the 12 analytic themes. These provide contextual support for the thematic synthesis described in the Results section.

**1. Knowledge, Attitudes, and Skills Gained**

## Theme 1. Knowledge Acquisition

*“By jotting down the specifics, we got to learn new names and google them... Some solid knowledge amassed that way.” (SFGD1 – Student 3)*

## Theme 2. Attitudinal Shifts

*“Now we have a chance to develop skills, including precision and teamwork. This cannot be accomplished by one person alone.” (SFGD1 – Student 5)*

*“And the responsibility! We each must take this seriously, or we start from scratch when one of us messes up somewhere. It teaches us responsibility (SFGD2 – Student 1).”*

## Theme 3. Skill Development

*“I was asked to draw charts and tables... I googled and studied the steps.” (SFGD2 – Student 1)*

*“All in all, what we’ve really gained is teamwork, plus the spirit and leadership of the teacher. (SFGD1 – Student 4)‬”*

**2. Challenges and Suggestions for Improvement**

## Theme 4. Student Participation

*“A group of 41 students is too large.” (SFGD1 – Student 6)*

*“Now they (students) have to devote to it large chunks of their private studying time. (FFGD1 – Teacher 2)”*

## Theme 5. Research Readiness

*“Microsoft Excel presented great problems... Some need for training there.” (SFGD1 – Student 6)
“The areas of study chosen for them (3^rd^ year medical students) are more often clinical, like cancers or COPD, only taught at Final Part 1 (4^th^ year medical students)…. making things difficult for them. (FFGD1 – Teacher 8)”*

## Theme 6. Financial Constraints

*“This is a (medical) school activity, the way I see it, related to all three departments. They should’ve proposed a budget, if only half, to the university if they have this RCA in mind.” (SFGD2 – Student 4)”*
*“If possible, a specific budget should be included under Third-MB Research Projects, whatever the grant is or from whichever level it may come, MOHS (Ministry of Health and Sports) or the rector himself. (FFGD1 – Teacher 2)”*

## Theme 7. Scheduling Conflicts

*“We prefer staying (working) on at the same ward while conducting (RCA) research.” (SFGD1 – Student 7)
“Include this (RCA) as a vertical module in the integrated curriculum.” (FFGD1 – Teacher 7)*

## Theme 8. Supervision Styles

*“The teachers revised a lot, some up to seven times!” (SFGD2 – Student 4)*

*“Teachers differ just as students differ, and there are some teachers who are not actively involved (in RCA). (FFGD2 – Teacher 7)”*

*“Every teacher is perfectly capable without a hierarchical structure of overseeing small projects that only aim to instill a research interest in students. I see no reason why it shouldn’t work to give the teachers more latitude. (FFGD2 – Teacher 6)”*

## Theme 9. Clinician Coordination

*“The hospital... should have a person charged with student affairs.” (SFGD1 – Student 7)
“… we have the clinical teachers proclaiming how their workload precludes them from participating. It happens. These teachers are, in fact, better able to track the data and would make more efficient (RCA) supervisors. (FFGD2 – Teacher 7)”*

## Theme 10. Data Management

*“They (patients)’re in and out of the hospital, staying month-long each time, hence the issue with our sample size; see, the forty of us were tasked with collecting three cases each, a total of 120 samples. Not nearly as many inpatients there, so to fill our quotas, some of us, including myself, ended up case-hunting at OPD. (SFGD2 – Student 8)”*

*“*

## Theme 11. RCA Event Logistics

*“Why not reserve these three or four days before the event for preparation, might I suggest?” (SFGD2 – Student 2)*

*“It’s good practice for students to question and answer one another (at RCA event). This has little place for the voice of the teacher.” (FFGD1 – Teacher 3)*

## Theme 12. Digitalization

*“They may simply share the e-books... All attendees (of RCA on the presentation-event day) should be able to receive a copy of the book, if desired.” (FFGD2 – Teacher 6)*
